# Efficacy and safety of parecoxib and flurbiprofen axetil for perioperative analgesia in children: a network meta-analysis

**DOI:** 10.3389/fmed.2023.1231570

**Published:** 2023-07-17

**Authors:** Xi Chen, Pan Chen, Xiao Chen, Min Huang, Kejing Tang, Qiuyi He

**Affiliations:** ^1^Department of Pharmacy, The First Affiliated Hospital, Sun Yat-sen University, Guangzhou, China; ^2^Institute of Clinical Pharmacology, School of Pharmaceutical Sciences, Sun Yat-sen University, Guangzhou, China

**Keywords:** NSAIDs, parecoxib, flurbiprofen axetil, children, perioperative analgesia

## Abstract

**Objective:**

The aim of this study was to systematically review the efficacy and safety of parecoxib and flurbiprofen axetil for perioperative analgesia in children through Bayesian network meta-analysis.

**Methods:**

We systematically searched PubMed, Embase, Cochrane Library, Web of Science, Sinomed, CNKI, VIP, and Wanfang Data databases on 18 July 2022 to obtain randomized controlled trials comparing perioperative parecoxib or flurbiprofen with placebo or standard treatment for pediatric analgesia. The outcomes were the postoperative pain score and the incidence of adverse events. The Gemtc package of R-4.0.3 and Stata 17.0 were used for Bayesian network meta-analysis.

**Results:**

We retrieved 942 articles and 49 randomized controlled trials involving 3,657 patients who met the inclusion criteria. Compared with children who received placebo treatment, those who received flurbiprofen axetil had lower pain sores at each time point within 24 h postoperatively, and those who received parecoxib had lower pain sores at each time point within 12 h postoperatively. Compared with children who received tramadol treatment, both the children who received flurbiprofen axetil or parecoxib had lower pain scores at 8 h postoperatively. The ranking results demonstrated that flurbiprofen axetil had significant superiority in reducing pain scores at 2, 4, and 12 h postoperatively, and parecoxib had significant superiority in reducing pain scores at 0, 0.5, 1, 6, 8, and 24 h postoperatively. In terms of safety, compared with children who received placebo, those who received flurbiprofen axetil or parecoxib had a lower incidence of total adverse events and postoperative agitation. Compared with tramadol, flurbiprofen axetil and parecoxib both significantly reduced the incidence of total adverse events and postoperative nausea and vomiting. Compared with flurbiprofen axetil or fentanyl, parecoxib significantly reduced the incidence of postoperative nausea and vomiting. The ranking results showed that parecoxib was advantageous in decreasing the incidence of total adverse events and postoperative nausea and vomiting.

**Conclusion:**

Flurbiprofen axetil was most effective at reducing pain scores at 2, 4, and 12 h postoperatively. Parecoxib had an advantage in terms of reducing pain scores at 0, 0.5, 1, 6, 8, and 24 h postoperatively, as well as the incidence of total adverse events and postoperative nausea and vomiting.

**Systematic trial registration:**

https://www.crd.york.ac.uk/PROSPERO/display_record.php?RecordID=348886, PROSPERO (CRD42022348886).

## 1. Introduction

Nearly half of the children still report moderate to severe pain during postoperative hospitalization (1). The results of a survey conducted in China showed that 42 out of 66 hospitals (63.64%) implemented pain management in children, but only eight (12.12%) achieved full coverage of pain assessment for children, including all outpatients and hospitalized children (2). Pain in early childhood that is inadequately treated, unrecognized, or poorly managed can persist into adulthood and lead to severe and persistent negative consequences (3). On the one hand, it can turn into chronic postoperative pain, which can cause functional impairment, disability, or depression (4). On the other hand, it may affect children's future healthcare experience, deepen unpleasant memories and psychological burdens, and generate anxiety and fear (5). Therefore, perioperative analgesia in children is essential.

There are limited options for analgesics for children. Ethical constraints in pediatric clinical trials have led to inadequate clinical evidence for many analgesics in children compared with adults, and the labels of most analgesics clearly state that they are not recommended for children (6). Opioids are still the cornerstone of multimodal analgesic regimens for children with moderate to severe acute pain, but their adverse events have been a frequent problem (7). The most common opioid complications, such as nausea, dizziness, and vomiting, occurred at the rates of 18.9, 18.4, and 13.4%, respectively (8). The incidence of pruritus caused by intravenous morphine injection ranges 2–10% (9). Therefore, a multimodal analgesic regimen based on non-steroidal anti-inflammatory drugs is recommended for effective perioperative pain control and the reduction of opioid consumption. Non-steroidal anti-inflammatory drugs are recommended as part of multimodal analgesia in the perioperative period in children by consensus and guidelines in several countries (10–12).

Flurbiprofen axetil is an injectable, non-selective cyclooxygenase (COX) inhibitor. Owing to the novel drug carrier system of lipid microspheres, flurbiprofen axetil can be targeted to inflammatory sites and surgical incisions and control the release of the encapsulated drug for a longer-lasting effect (13). Flurbiprofen axetil also readily penetrates into the cerebrospinal fluid, where it can reach concentrations of up to seven times higher than unconjugated plasma concentrations, contributing to the analgesic effect in the central nervous system (14). Parecoxib is the first injectable selective COX-2 inhibitor that is rapidly converted to its active metabolite, valdecoxib, after hepatic enzymatic hydrolysis *in vivo* (15). Parecoxib has a highly selective inhibitory effect on COX-2 and reduces peripheral and central sensitization by blocking the arachidonic acid cascade and inhibiting prostaglandin synthesis, thus providing an analgesic effect. It has no inhibitory effect on COX-1 at the recommended dose and has little impact on the normal physiological function of the gastric mucosa and platelets (16).

Clinical evidence for the use of parecoxib and flurbiprofen ester for perioperative analgesia in children has emerged as multimodal analgesia regimens and is increasingly advocated. However, only one meta-analysis published in 2015 systematically evaluated the effect of perioperative parecoxib on postoperative pain in children, demonstrating that, compared with placebo, parecoxib reduced pain scores and the incidence of adverse events (17). The efficacy and safety of flurbiprofen axetil in children remain unclear, and few studies have directly compared the drugs. Therefore, we performed a Bayesian network meta-analysis that integrated as much data as possible from direct and indirect comparative evidence to systematically evaluate the efficacy and safety of parecoxib and flurbiprofen axetil in pediatric perioperative analgesia.

## 2. Methods

### 2.1. Search strategy

This network meta-analysis was registered at PROSPERO (CRD42022348886), and the work was conducted following the guidelines of the Preferred Reporting Items for Systematic Reviews and Meta-analysis (PRISMA) Statement. We systematically searched PubMed, Embase, Cochrane Library, Web of Science, Sinomed, CNKI, VIP, and Wanfang Data databases on 18 July 2022, using “parecoxib,” “flurbiprofen axetil,” “child, preschool,” “adolescent,” “postoperative period,” “perioperative period,” “intraoperative period,” “preoperative period,” “surgical procedures,” “operative,” and “general surgery” as keywords and MeSH terms combined with Boolean operators. The search equations can be scrutinized in Supplementary Appendix S1. Two reviewers independently screened all titles and abstracts, excluded duplicate studies, and selected works for final analysis via full-text analysis. Any disagreements were resolved by discussion with the third author.

### 2.2. Inclusion and exclusion criteria

The included studies were limited to those published in English or Chinese. The participants were children or adolescents (younger than 18 years) who underwent elective surgery with general anesthesia. The study groups received parecoxib or flurbiprofen axetil, and the control groups received placebo or standard treatment (fentanyl or tramadol) for perioperative analgesia. The outcomes evaluated were postoperative pain at 0, 0.5, 1, 2, 4, 6, 8, 12, and 24 h and the incidence of total adverse events, nausea and vomiting, and agitation. We excluded guidelines, reviews, comments, conference abstracts without full text and nonrandomized controlled studies.

### 2.3. Data extraction

Two reviewers extracted the data independently. Disagreements were resolved by consensus through discussion and consultation with a third reviewer. The name of the first author, year of publication, sample size, patient age, surgery type, study design, and outcome measures were extracted from each study. In multi-arm trials, we only extracted the data from the arm related to our research.

### 2.4. Quality assessment

The quality of the included articles was evaluated by two reviewers using the Cochrane risk-of-bias tool for randomized controlled trials (RCTs) in the following domains: random sequence generation, concealment of allocation sequence, blinding of patients or outcome assessors, incomplete outcome data, selective reporting, and other types of bias. Disagreements were resolved through discussion or by the third reviewer. The risk of bias in each RCT was graded as high, low, or unclear.

### 2.5. Statistical analysis

The gemtc package of R 4.0.3, based on the Markov Chain Monte Carlo method, was used for Bayesian network meta-analysis. The mean difference with 95% confidence intervals (CIs) was used as the treatment effect estimator for continuous variables, while for dichotomous variables, it was the odds ratio. An MD of <0 indicated that the experimental group had a smaller value. The 95% CIs that did not include 0 were considered to show a significant difference between the two groups, which were compared. The odds ratio of <1 indicated that the incidence in the experimental group was lower. The 95% CIs that did not include 1 were considered to show a significant difference between the two groups. Global heterogeneity was assessed by *I*^2^ and of >50% indicated significant heterogeneity. We used the node-splitting analysis to evaluate the inconsistency between the direct and indirect comparisons. If the *P*-value was >0.05, it indicated that direct and indirect comparisons tended to be consistent. We used random-effect consistency models within a Bayesian framework. The potential scale reduction factor proposed by Brooks et al. was used to diagnose the convergence degree of the model, and if the value tended to 1, the convergence was good (18). We plotted the ranking probability diagrams based on the surface under the cumulative ranking curve. Funnel plots were generated by Stata 17.0 software to evaluate the publication bias of outcome indicators.

## 3. Results

### 3.1. Study selection and characteristics

**Figure 1:**
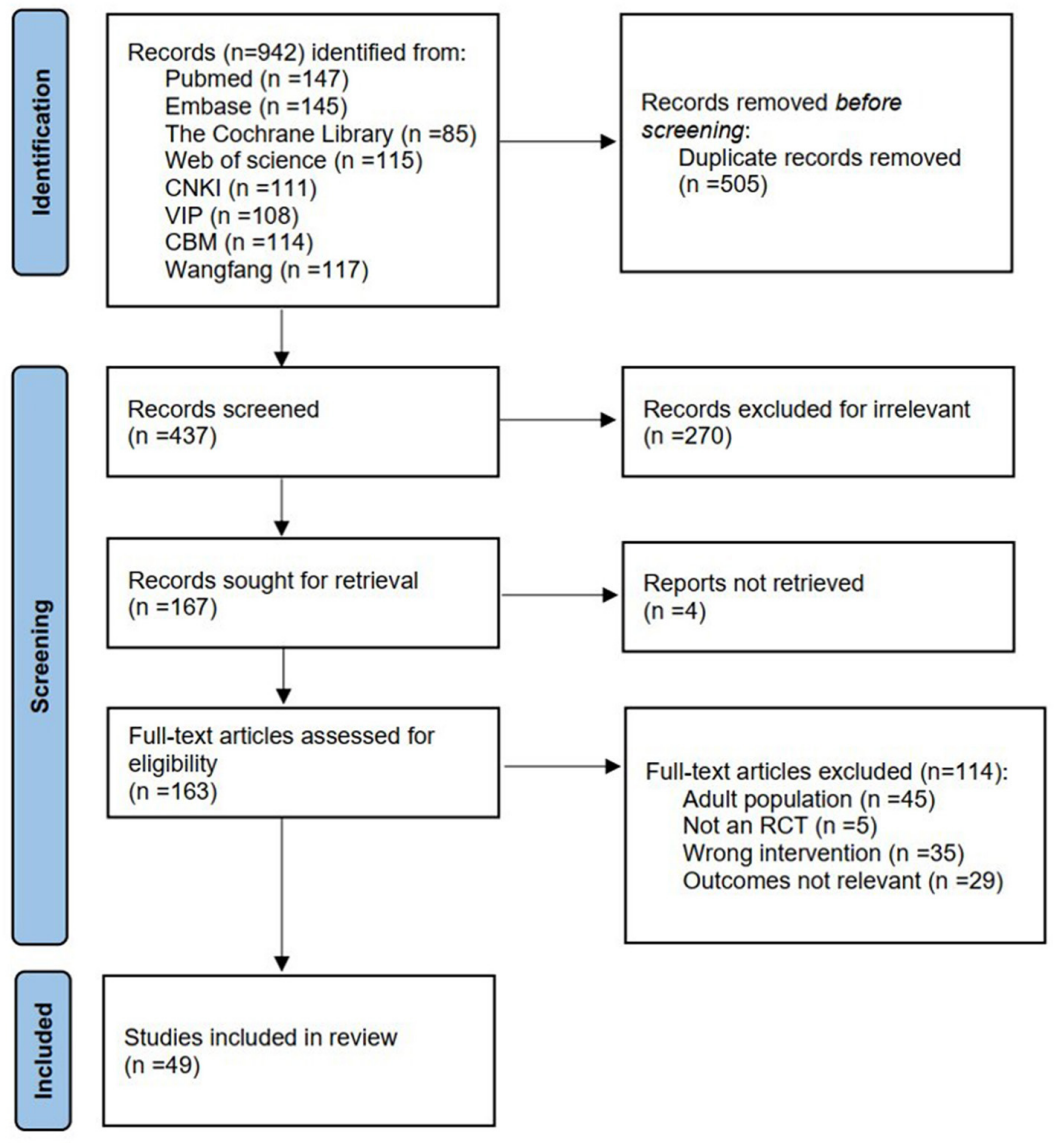
PRISMA flowchart of the study.

We retrieved 942 articles, and after the duplicates were removed, 437 articles were screened through titles and abstracts, and the full texts of 167 articles were retrieved to assess eligibility. A total of 49 RCTs involving 3,657 patients were included in the final analysis (19–67). The PRISMA flow diagram is shown in Figure 1.

The characteristics of the included studies are shown in Supplementary Table 1. A total of 23 RCTs studied tonsillectomy/adenoidectomy; 14 studied abdominal surgery/laparoscopic surgery; and three each studied plastic surgery, circumcision, strabismus surgery, and orthopedic surgery. The following interventions were compared in these RCTs: 30 compared flurbiprofen axetil with placebo; 5 flurbiprofen axetil with fentanyl; 7 flurbiprofen axetil with tramadol; 1 flurbiprofen axetil with parecoxib; 13 parecoxib with placebo; 2 parecoxib with fentanyl; and 6 parecoxib with tramadol.

### 3.2. Assessment of study quality

**Figure 2:**
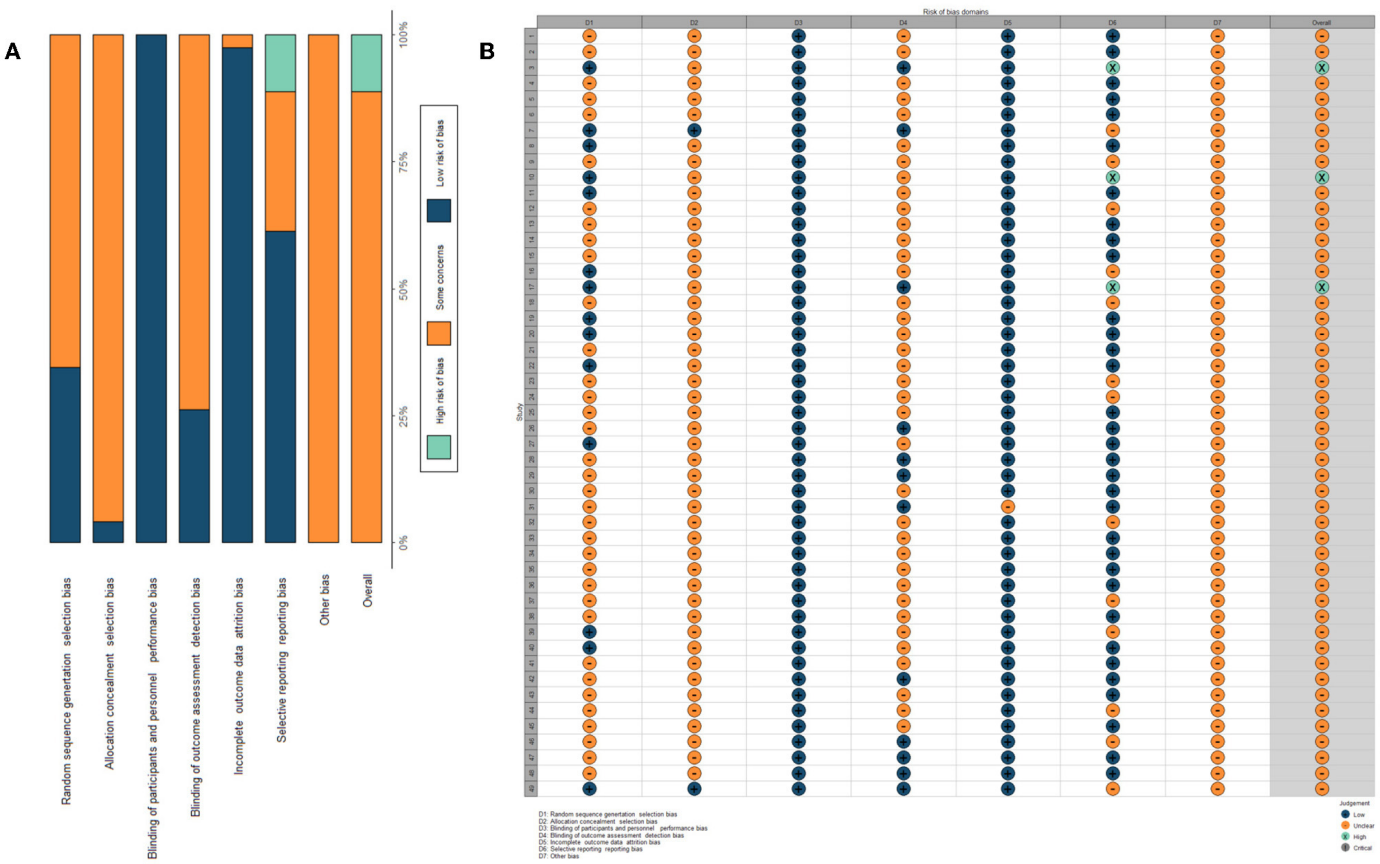
Assessment of the risk of bias. **(A)** Risk of bias summary table: it is a summary table of review authors' judgments for each risk of bias entry for each study. **(B)** Assessment of risk of bias within each trial: it is the distribution of judgments (low, high, and unclear) across studies for each risk of bias entry.

The quality assessment results of the included RCTs are summarized in Figure 2. The overall study quality grading on individual parameters was low. A total of 12 articles described specific methods of random sequence generation: only 2 claimed allocation concealment and nine blinded the participants or evaluators. A total of 3 RCTs were evaluated as having a high risk of bias, and 46 were identified as having an unclear risk of bias.

### 3.3. Postoperative pain scores

**Figure 3:**
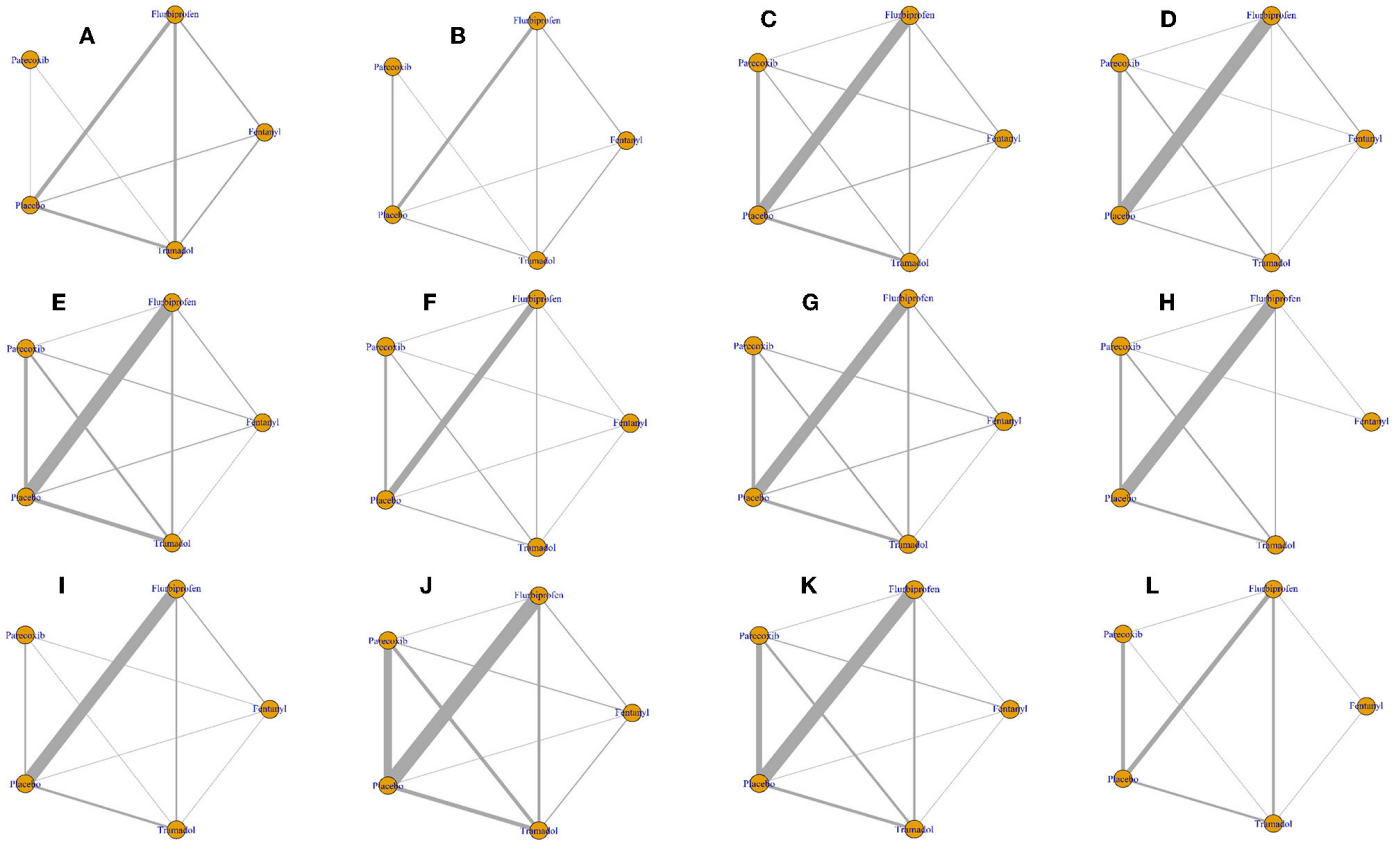
Network plot of all outcomes. The nodes represent the drugs involved, and the size of every node is proportional to the number of randomized participants. The lines between the nodes represent direct comparisons between drugs, and the width of the lines indicates the cumulative number of RCTs for each pairwise comparison. **(A)** Pain scores at postoperative 0 h. **(B)** Pain scores at postoperative 0.5 h. **(C)** Pain scores at postoperative 1 h. **(D)** Pain scores at postoperative 2 h. **(E)** Pain scores at postoperative 4 h. **(F)** Pain scores at postoperative 6 h. **(G)** Pain scores at postoperative 8 h. **(H)** Pain scores at postoperative 12 h. **(I)** Pain scores at postoperative 24 h. **(J)** Incidence of total adverse events. **(K)** Incidence of postoperative nausea and vomiting. **(L)** Incidence of agitation after surgery.

A total of 9 RCTs reported pain scores at postoperative 0 and 0.5 h and 8 direct comparisons were established in the network model; 27 trials reported pain scores at postoperative 1 h and 10 direct comparisons were established in the network model; 30 trials reported pain scores at postoperative 2 h and 10 direct comparisons were established in the network model; 31 trials reported pain scores at postoperative 4 h and 10 direct comparisons were established in the network model; 17 trials reported pain scores at postoperative 6 h and 10 direct comparisons were established in the network model; 28 trials reported pain scores at postoperative 8 h and 9 direct comparisons were established in the network model; 28 trials reported pain scores at postoperative 12 h and 8 direct comparisons were established in the network model; and 26 trials reported pain scores at postoperative 24 h and 9 direct comparisons were established in the network model (Figure 3).

**Figure 4:**
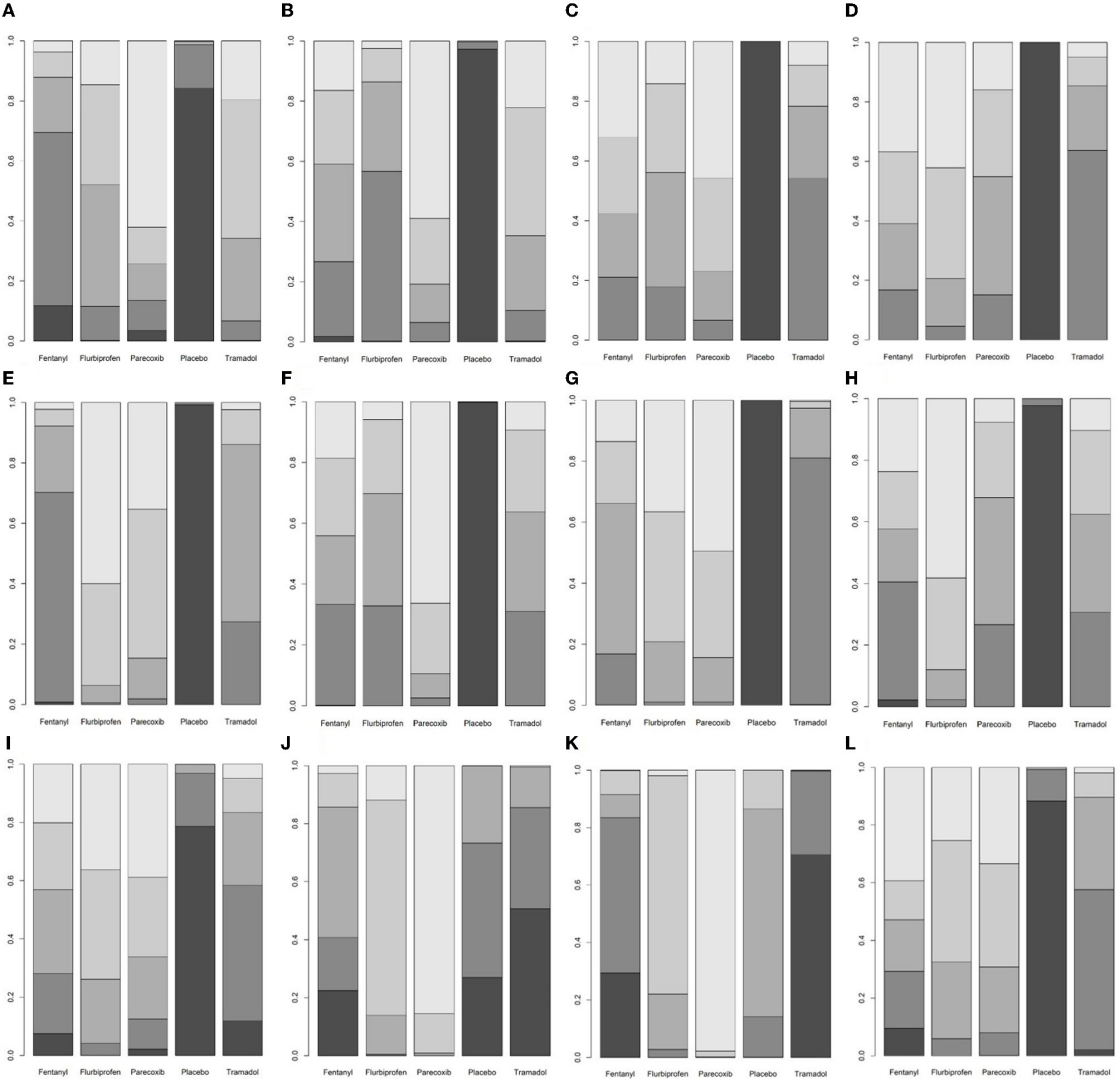
A ranking plot based on the probabilities of drugs. The horizontal axis shows the different interventions, and the vertical axis shows the ranking probability. The lighter the color of the block, the higher the ranking, and the darker the color of the block, the lower the ranking. **(A)** Pain scores at postoperative 0 h. **(B)** Pain scores at postoperative 0.5 h. **(C)** Pain scores at postoperative 1 h. **(D)** Pain scores at postoperative 2 h. **(E)** Pain scores at postoperative 4 h. **(F)** Pain scores at postoperative 6 h. **(G)** Pain scores at postoperative 8 h. **(H)** Pain scores at postoperative 12 h. **(I)** Pain scores at postoperative 24 h. **(J)** Incidence of total adverse events. **(K)** Incidence of postoperative nausea and vomiting. **(L)** Incidence of agitation after surgery.

At postoperative 0 h, flurbiprofen axetil and tramadol reduced the pain scores compared with placebo (Table 1). At postoperative 0.5, 1, 2, 4, 6, 8, and 12 h, compared with placebo, fentanyl, flurbiprofen axetil, parecoxib, and tramadol all reduced the pain scores (Table 1). At postoperative 24 h, the pain score of the flurbiprofen axetil group was significantly lower than that of the placebo group (Table 1). Compared with the tramadol group, the postoperative 8-h pain scores of the flurbiprofen axetil or parecoxib groups were significantly lower (Table 1). The ranking results demonstrated that flurbiprofen axetil had significant superiority in reducing pain scores at 2, 4, and 12 h postoperatively, and parecoxib was the highest-ranking drug to reduce pain scores at 0, 0.5, 1, 6, 8, and 24 h postoperatively (Supplementary Table 3, Figure 4).

**Table 1:** Network meta-analysis of postoperative pain scores.

		**Fentanyl**	**Flurbiprofen**	**Parecoxib**	**Placebo**
Pain scores at postoperative 0 h	Flurbiprofen	0.87 (−1.08, 2.83)			
	Parecoxib	1.77 (−1.93, 5.44)	0.90 (−2.53, 4.29)		
	Placebo	−1.13 (−3.16, 0.89)	**−2.00 (−3.36**, **−0.64)**	−2.89 (−6.16, 0.40)	
	Tramadol	1.05 (−0.92, 3.05)	0.18 (−1.35, 1.73)	−0.71 (−3.95, 2.58)	**2.18 (0.66, 3.72)**
Pain scores at postoperative 0.5 h	Flurbiprofen	−0.55 (−3.03, 1.93)			
	Parecoxib	0.99 (−2.23, 4.18)	1.55 (−1.02, 4.05)		
	Placebo	**−2.74 (−5.30**, **−0.17)**	**−2.18 (−3.69**, **−0.70)**	**−3.73 (−5.86**, **−1.56)**	
	Tramadol	0.39 (−2.16, 2.95)	0.94 (−1.22, 3.11)	−0.61 (−3.28, 2.12)	**3.12 (0.99, 5.27)**
Pain scores at postoperative 1 h	Flurbiprofen	−0.15 (−1.63, 1.33)			
	Parecoxib	0.20 (−1.32, 1.71)	0.34 (−0.83, 1.52)		
	Placebo	**−3.02 (−4.48**, **−1.58)**	**−2.88 (−3.55**, **−2.20)**	**−3.22 (−4.27**, **−2.17)**	
	Tramadol	−0.47 (−2.20, 1.24)	−0.33 (−1.59, 0.93)	−0.67 (−2.08, 0.74)	**2.55 (1.36, 3.75)**
Pain scores at postoperative 2 h	Flurbiprofen	0.10 (−1.02, 1.21)			
	Parecoxib	−0.14 (−1.35, 1.06)	−0.24 (−1.07, 0.60)		
	Placebo	**−2.81 (−3.94**, **−1.70)**	**−2.91 (−3.34**, **−2.48)**	**−2.67 (−3.44**, **−1.90)**	
	Tramadol	−0.51 (−1.82, 0.80)	−0.61 (−1.64, 0.43)	−0.37 (−1.35, 0.62)	**2.30 (1.30, 3.31)**
Pain scores at postoperative 4 h	Flurbiprofen	0.99 (−0.14, 2.10)			
	Parecoxib	0.85 (−0.29, 1.98)	−0.14 (−0.97, 0.71)		
	Placebo	**−1.37 (−2.47**, **−0.27)**	**−2.35 (−2.83**, **−1.88)**	**−2.21 (−2.98**, **−1.45)**	
	Tramadol	0.34 (−0.87, 1.55)	−0.65 (−1.45, 0.15)	−0.51 (−1.38, 0.36)	**1.70 (0.95, 2.45)**
Pain scores at postoperative 6 h	Flurbiprofen	−0.08 (−1.50, 1.33)			
	Parecoxib	0.56 (−0.91, 2.02)	0.64 (−0.42, 1.69)		
	Placebo	**−2.27 (−3.67**, **−0.88)**	**−2.19 (−2.81**, **−1.57)**	**−2.83 (−3.81**, **−1.85)**	
	Tramadol	−0.05 (−1.55, 1.45)	0.04 (−1.07, 1.14)	−0.60 (−1.75, 0.54)	**2.22 (1.15, 3.31)**
Pain scores at postoperative 8 h	Flurbiprofen	0.31 (−0.68, 1.30)			
	Parecoxib	0.39 (−0.62, 1.39)	0.07 (−0.75, 0.89)		
	Placebo	**−1.71 (−2.69**, **−0.76)**	**−2.03 (−2.48**, **−1.58)**	**−2.10 (−2.83**, **−1.35)**	
	Tramadol	−0.52 (−1.63, 0.59)	**−0.83 (−1.65**, **−0.01)**	**−0.91 (−1.78**, **−0.02)**	**1.19 (0.43, 1.97)**
Pain scores at postoperative 12 h	Flurbiprofen	0.43 (−0.96, 1.84)			
	Parecoxib	0.00 (−1.39, 1.39)	−0.43 (−1.25, 0.37)		
	Placebo	**−1.45 (−2.86,−0.05)**	**−1.89 (−2.31**, **−1.47)**	**−1.46 (−2.20**, **−0.70)**	
	Tramadol	0.01 (−1.52, 1.54)	−0.42 (−1.26, 0.41)	0.01 (−0.86, 0.89)	**1.47 (0.67, 2.27)**
Pain scores at postoperative 24 h	Flurbiprofen	0.18 (−0.67, 1.04)			
	Parecoxib	0.17 (−0.80, 1.14)	−0.01 (−0.84, 0.80)		
	Placebo	−0.58 (−1.44, 0.28)	**−0.77 (−1.11**, **−0.43)**	−0.76 (−1.52, 0.03)	
	Tramadol	−0.21 (−1.19, 0.77)	−0.40 (−1.08, 0.28)	−0.39 (−1.31, 0.55)	0.37 (−0.30, 1.04)

The bold values indicate that significant differences between two treatments.

### 3.4. Incidence of adverse events

A total of 40 RCTs reported the incidence of total adverse events and 10 direct comparisons were established in the network model; 34 trials reported the incidence of postoperative nausea and vomiting and 10 direct comparisons were established in the network model; and 13 trials reported the incidence of postoperative agitation and 7 direct comparisons were established in the network model (Figure 3).

Compared with the placebo group, flurbiprofen axetil and parecoxib reduced the incidence of total adverse events and postoperative agitation (Table 2). Compared with the tramadol group, flurbiprofen axetil and parecoxib reduced the incidence of total adverse events and postoperative nausea and vomiting (Table 2). The incidence of postoperative nausea and vomiting was significantly lower in the parecoxib group than in the flurbiprofen axetil, fentanyl, and placebo groups (Table 2). There was no significant difference between the other comparisons. The ranking results showed that parecoxib was relatively more advantageous in decreasing the incidence of total adverse events and postoperative nausea and vomiting (Supplementary Table 3, Figure 4).

**Table 2:** Network meta-analysis of the incidence of adverse events.

		**Fentanyl**	**Flurbiprofen**	**Parecoxib**	**Placebo**
Incidence of total adverse events	Flurbiprofen	1.71 (0.64, 4.46)			
	Parecoxib	2.48 (0.96, 6.37)	1.46 (0.77, 2.82)		
	Placebo	0.84 (0.33, 2.1)	**0.49 (0.32, 0.77)**	**0.34 (0.2, 0.57)**	
	Tramadol	0.76 (0.28, 1.93)	**0.45 (0.23, 0.83)**	**0.31 (0.16, 0.57)**	0.9 (0.5, 1.57)
Incidence of postoperative nausea and vomiting	Flurbiprofen	1.9 (0.76, 4.86)			
	Parecoxib	**3.5 (1.5, 8.99)**	**1.87 (1.03, 3.39)**		
	Placebo	1.59 (0.64, 3.77)	0.84 (0.59, 1.18)	**0.45 (0.26, 0.74)**	
	Tramadol	0.77 (0.31, 1.92)	**0.41 (0.22, 0.74)**	**0.22 (0.12, 0.39)**	**0.49 (0.28, 0.82)**
Incidence of agitation after surgery	Flurbiprofen	1.07 (0.07, 13.3)			
	Parecoxib	1.13 (0.06, 14.97)	1.06 (0.28, 3.35)		
	Placebo	0.2 (0.01, 2.33)	**0.19 (0.07, 0.41)**	**0.18 (0.07, 0.43)**	
	Tramadol	0.56 (0.04, 6.15)	0.52 (0.14, 1.58)	0.49 (0.13, 1.81)	2.75 (0.99, 8.36)

The bold values indicate that significant differences between two treatments.

### 3.5. Exploration of consistency, convergence, heterogeneity, and publication bias

Across all outcomes, the assessment of inconsistency always supported the assumption of consistency between direct and indirect evidence. The results of the node splitting models are shown in Supplementary Figure 1. The potential scale reduction factor (PSRF) was used to diagnose the convergence degree of the model and the PSRF value tends to 1 indicates that the convergence is good (Supplementary Figure 2). The global heterogeneity and pairwise comparisons of heterogeneity are shown in Supplementary Figure 3. The comparison-adjusted funnel plots of all outcomes are shown in Supplementary Figure 4, and figures with poor symmetry indicate the possibility of publication bias.

## 4. Discussion

Our analysis included 49 RCTs with 3,657 children aged 6 months to 14 years, and the aim of this study was to systematically evaluate the efficacy and safety of parecoxib and flurbiprofen axteil in perioperative analgesia in children using a Bayesian network meta-analysis. The results demonstrated that the pain scores of the flurbiprofen axetil group were significantly lower than those in the placebo group at all time points in the 24-h postoperative period, and the pain scores at all time points during the 12-h postoperative period of the parecoxib group were significantly lower than those in the placebo group. From the potential rankings, flurbiprofen axetil was superior to other drugs in reducing pain scores at 2, 4, and 12 h postoperatively, while parecoxib had some advantages in reducing pain scores at 0, 0.5, 1, 6, 8, and 24 h postoperatively.

Few meta-analyses have been conducted to investigate the efficacy and safety of parecoxib or flurbiprofen axetil for perioperative analgesia in children, but evidence in adults is constantly being updated. A previous meta-analysis by Li et al. (68) reported that low to moderate evidence indicated that parecoxib relieved postoperative orthopedic pain while sparing opioid analgesic consumption without increasing the incidence of adverse events (68). Sun et al. (69) reported that the perioperative administration of flurbiprofen was effective in reducing postoperative pain, nausea, and vomiting in Chinese surgical patients (69). Another meta-analysis comparing flurbiprofen axetil vs. parecoxib found that parecoxib had an advantage over flurbiprofen axetil for patients with postoperative pain beyond 6 h (70). It has been reported that intravenous administration of parecoxib (1.0 mg/kg to a maximum of 40 mg) in children resulted in free valdecoxib concentrations above the IC_50_ for COX-2 for at least 12 h, and the area under the valdecoxib concentration-time curve was similar to that in adults (40 mg) (71). A population pharmacokinetic study of intravenous flurbiprofen axetil in Chinese patients with postoperative pain discovered that between-subject variability for flurbiprofen was associated with body height and weight (72). In children aged 3 months to 13 years, the estimated clearance rate was 0.96 L/h/70 kg and the volume of distribution at steady state was 8.1 L/70 kg, which was significantly lower than that in adults (14). Therefore, the efficacy and safety of parecoxib and flurbiprofen axetil for perioperative analgesia in children need to be confirmed, as the results of the adult studies cannot be directly extrapolated to children because of the limitations of individual differences between adults and children.

In a previous network meta-analysis, parecoxib for analgesia after tonsillectomy in children was found to be better than fentanyl, and flurbiprofen axetil was superior to tramadol (73), but it only reported one pain score and did not specify the pain scores in children at different time points. Another meta-analysis found that the perioperative use of parecoxib in children was associated with less acute postoperative pain and adverse events compared with placebo or standard treatment (fentanyl or tramadol) (17), but that study lacked an evaluation of the analgesic effects of parecoxib beyond 12 h postoperatively. Our network meta-analysis systematically evaluated the efficacy and safety of parecoxib and flurbiprofen axetil for perioperative analgesia in children while adding more recent studies to provide a more reliable evidence-based analysis.

In terms of safety, compared with children who received placebo, those who received flurbiprofen axetil or parecoxib had a lower incidence of total adverse events and postoperative agitation. Compared with tramadol, flurbiprofen axetil and parecoxib both significantly reduced the incidence of total adverse events and postoperative nausea and vomiting. Compared with flurbiprofen axetil or fentanyl, parecoxib significantly reduced the incidence of postoperative nausea and vomiting. The ranking results showed that parecoxib was more advantageous in decreasing the incidence of total adverse events and postoperative nausea and vomiting.

Because few adverse events were reported in the included trials, such as respiratory depression, pruritus, headache, dizziness, and urinary retention, only total adverse events, postoperative nausea and vomiting, and agitation were analyzed in this study because of insufficient data. A multicenter retrospective study involving 3,542 patients found that postoperative administration of parecoxib resulted in a 0.2% incidence of adverse events, primarily hypotension, nausea, vomiting, pruritus, and rash (74). Another study discovered that flurbiprofen axetil accounted for the largest number of adverse reactions (686/5597, 12.26%), with the most common being nausea, rash, vomiting, pruritus, and dizziness (75). Several studies have revealed that, compared with tramadol, parecoxib and flurbiprofen axetil both resulted in less postoperative nausea and vomiting in adults (76–78). Our study also found in children that the incidence of postoperative nausea and vomiting was lower for both parecoxib and flurbiprofen axetil than for tramadol. Bu et al. (17) found that, when compared to placebo or standard treatment, perioperative parecoxib administration for children resulted in a lower incidence of postoperative nausea, vomiting, and agitation (17). Li et al. (67) also discovered that the incidence of postoperative nausea and vomiting was significantly higher in the placebo group (11/30, 37%) than in the parecoxib group (4/30, 13%) (67). These results suggested that parecoxib was an effective and safe analgesic for children, particularly in reducing postoperative nausea and vomiting, which is consistent with the results of our study. The included trials demonstrated that none of the children had bleeding, gastrointestinal injury, renal injury, or cardiovascular events. In a pooled analysis of 28 randomized, double-blind, placebo-controlled clinical trials, patients in the parecoxib and placebo groups had similar frequencies of gastrointestinal events, renal failure and impairment, and cardiovascular embolic and thrombotic events (79). Therefore, this study considered that both parecoxib and flurbiprofen axetil decreased the risk of total adverse events compared with placebo and tramadol and that parecoxib was more advantageous in reducing postoperative nausea and vomiting.

There were a few limitations to this study. First, the included trials were associated with high risks of bias because most of them only mentioned “random” without specifying the method of generating the random sequence or describing the allocation concealment in detail. Second, most studies only recorded pain scores within the first 24 h after surgery, paying little attention to 48 or 72 h. Furthermore, we did not analyze other outcomes, such as opioid consumption or proportions of patients requiring rescue analgesia, which may have more clinical implications. Third, data on direct head-to-head comparisons of flurbiprofen axteil and parecoxib within the network were insufficient. Finally, there were some differences between studies in the type of surgery, perioperative care protocol, study drug dose, and timing of administration, which could explain the heterogeneity in our results.

## 5. Conclusion

Based on the available clinical evidence, our network meta-analysis reached the following conclusions. Perioperative administration of flurbiprofen axetil and parecoxib were both effective in reducing postoperative pain scores and total adverse events compared with placebo. Flurbiprofen axetil and parecoxib also caused fewer total adverse events and postoperative nausea and vomiting compared with tramadol. Flurbiprofen axetil was most effective at reducing pain scores at 2, 4, and 12 h postoperatively. Parecoxib had an advantage in terms of reducing pain scores at 0, 0.5, 1, 6, 8, and 24 h postoperatively, as well as reducing the incidence of total adverse events and postoperative nausea and vomiting.

## Data availability statement

The original contributions presented in the study are included in the article/Supplementary material, further inquiries can be directed to the corresponding author.

## Author contributions

XiC helped with study design, the selection of included articles, data extraction, quality assessment, data analysis and interpretation, and writing the manuscript. PC and QH helped with the study design, selection of included articles, data extraction, quality assessment, data analysis and interpretation, and the final review. XiaC, MH, and KT helped with the study design and the final review. All authors contributed to the article and approved the submitted version.
