# Supplementary material for: Efficacy and safety of parecoxib and flurbiprofen axetil for perioperative analgesia in children: a network meta-analysis
Source: Front Med (Lausanne). 2023 Jul 17;10:1231570. doi: 10.3389/fmed.2023.1231570 (PMC10387543; doi:10.3389/fmed.2023.1231570)

***Supplementary Material***

**Efficacy and safety of parecoxib and flurbiprofen axetil for perioperative analgesia in children: A network meta-analysis**

Xi Chen^1,2^, Pan Chen^1^, Xiao Chen^1^, Min Huang^2^, Kejing Tang^1^, Qiuyi He^1*^

1 Department of Pharmacy, The First Affiliated Hospital, Sun Yat-sen University, Guangzhou, China

2 Institute of Clinical Pharmacology, School of Pharmaceutical Sciences, Sun Yat-sen University, Guangzhou, China

*** Correspondence:**

Corresponding Author: Qiuyi He

E-mail: [sysu_h@163.com](mailto:sysu_h@163.com)

**Contents**

[Supplemental Table 1: Search equations. 2](#_Toc135215861)

[Supplemental Table 2: The characteristics of included studies. 4](#_Toc135215862)

[Supplemental Table 3: Ranking results of all outcomes. 6](#_Toc135215863)

[Supplemental Figure 1: Results of Node-splitting analysis. 10](#_Toc135215864)

[Supplemental Figure 2: Results of convergence analysis. 13](#_Toc135215865)

[Supplemental Figure 3: The pairwise comparisons of heterogeneity. 14](#_Toc135215866)

[Supplemental Figure 4: The funnel plot of enrolled trials. 17](#_Toc135215867)

Supplemental Table 1: Search equations.

| **Number** | **PubMed** |
| --- | --- |
| 1 | (((flurbiprofen axetil[Supplementary Concept]) OR (Flurbiprofen[MeSH Terms])) OR (((flurbiprofen axetil[Title/Abstract]) OR (Flurbiprofen[Title/Abstract])) OR (LFP83[Title/Abstract]))) |
| 2 | (((((((parecoxib[Title/Abstract]) OR (Isoxazoles[Title/Abstract])) OR (N-(((5-methyl-3-phenylisoxazol-4-yl)-phenyl)sulfonyl)propanamide[Title/Abstract])) OR (N-(((Me-P)-P)S)P[Title/Abstract])) OR (parecoxib sodium[Title/Abstract])) OR (N-(((5-methyl-3-phenylisoxazol-4-yl)-phenyl)sulfonyl)propanamine, sodium salt[Title/Abstract])) OR (Dynastat[Title/Abstract])) OR ((parecoxib[Supplementary Concept]) OR (isoxazoles[MeSH Terms])) |
| 3 | 1 OR 2 |
| 4 | ((((((((((((((Child, Preschool[Title/Abstract]) OR (Child[Title/Abstract])) OR (Adolescent[Title/Abstract])) OR (Preschool Child[Title/Abstract])) OR (Children, Preschool[Title/Abstract])) OR (Preschool Children[Title/Abstract])) OR (Children[Title/Abstract])) OR (Adolescen*[Title/Abstract])) OR (Teen*[Title/Abstract])) OR (Youth*[Title/Abstract])) OR (Adolescent*, Female[Title/Abstract])) OR (Female Adolescent*[Title/Abstract])) OR (Adolescent*, Male[Title/Abstract])) OR (Male Adolescent*[Title/Abstract])) OR (((Child, Preschool[MeSH Terms]) OR (Child[MeSH Terms])) OR (Adolescent[MeSH Terms])) |
| 5 | (((((((((((((((((((((((Postoperative Period*[Title/Abstract]) OR (Perioperative Period*[Title/Abstract])) OR (intraoperative Period*[Title/Abstract])) OR (preoperative Period[Title/Abstract])) OR (Period*, Postoperative[Title/Abstract])) OR (Period*, Perioperative[Title/Abstract])) OR (Period*, Intraoperative[Title/Abstract])) OR (Period, Preoperative[Title/Abstract])) OR (Surgical Procedures, Operative[Title/Abstract])) OR (General Surgery[Title/Abstract])) OR (Operative Procedure*[Title/Abstract])) OR (Procedure*, Operative[Title/Abstract])) OR (Surgical Procedure, Operative[Title/Abstract])) OR (Operative Surgical Procedures[Title/Abstract])) OR (Procedure*, Operative Surgical[Title/Abstract])) OR (Surgical Procedures[Title/Abstract])) OR (Procedure*, Surgical[Title/Abstract])) OR (Surgical Procedure[Title/Abstract])) OR (Operative Surgical Procedure[Title/Abstract])) OR (Surgery, Ghost[Title/Abstract])) OR (Ghost Surgery[Title/Abstract])) OR (Surgery, General[Title/Abstract])) OR (Surgery[Title/Abstract])) OR ((((((Postoperative Period[MeSH Terms]) OR (Perioperative Period[MeSH Terms])) OR (intraoperative Period[MeSH Terms])) OR (preoperative Period[MeSH Terms])) OR (Surgical Procedures, Operative[MeSH Terms])) OR (General Surgery[MeSH Terms])) |
| 6 | 3 AND 4 AND 5 |
| **Number** | **Embase** |
| 1 | 'flurbiprofen axetil'/exp OR 'flurbiprofen axetil' OR 'flurbiprofen'/exp OR 'flurbiprofen' |
| 2 | 'flurbiprofen':ab,ti OR 'flurbiprofen axetil':ab,ti OR 'lfp83':ab,ti |
| 3 | 'parecoxib'/exp |
| 4 | 'parecoxib':ab,ti OR 'parecoxib sodium':ab,ti OR 'dynastat':ab,ti |
| 5 | 1 OR 2 OR 3 OR 4 |
| 6 | 'preschool child'/exp OR 'child'/exp OR 'adolescent'/exp |
| 7 | 'child, preschool':ab,ti OR 'child':ab,ti OR 'adolescent':ab,ti OR 'preschool child':ab,ti OR 'children, preschool':ab,ti OR 'preschool children':ab,ti OR 'children':ab,ti OR 'adolescence':ab,ti OR 'adolescents':ab,ti OR 'teens':ab,ti OR 'teen':ab,ti OR 'teenagers':ab,ti OR 'teenager':ab,ti OR 'youth':ab,ti OR 'youths':ab,ti OR 'adolescents, female':ab,ti OR 'adolescent, female':ab,ti OR 'female adolescent':ab,ti OR 'female adolescents':ab,ti OR 'adolescents, male':ab,ti OR 'adolescent, male':ab,ti OR 'male adolescent':ab,ti OR 'male adolescents':ab,ti |
| 8 | 6 OR 7 |
| 9 | 'postoperative period'/exp OR 'perioperative period'/exp OR 'intraoperative period'/exp OR 'preoperative period'/exp OR 'surgery'/exp |
| 10 | 'postoperative period':ab,ti OR 'perioperative period':ab,ti OR 'intraoperative period':ab,ti OR 'preoperative period':ab,ti OR 'surgical procedures, operative':ab,ti OR 'general surgery':ab,ti OR 'period, postoperative':ab,ti OR 'periods, postoperative':ab,ti OR 'postoperative periods':ab,ti OR 'period, perioperative':ab,ti OR 'periods, perioperative':ab,ti OR 'perioperative periods':ab,ti OR 'intraoperative periods':ab,ti OR 'period, intraoperative':ab,ti OR 'periods, intraoperative':ab,ti OR 'period, preoperative':ab,ti OR 'operative procedures':ab,ti OR 'operative procedure':ab,ti OR 'procedure, operative':ab,ti OR 'procedures, operative':ab,ti OR 'surgical procedure, operative':ab,ti OR 'operative surgical procedures':ab,ti OR 'procedure, operative surgical':ab,ti OR 'procedures, operative surgical':ab,ti OR 'surgical procedures':ab,ti OR 'procedure, surgical':ab,ti OR 'procedures, surgical':ab,ti OR 'surgical procedure':ab,ti OR 'operative surgical procedure':ab,ti OR 'surgery, ghost':ab,ti OR 'ghost surgery':ab,ti OR 'surgery, general':ab,ti OR 'surgery':ab,ti |
| 11 | 9 OR 10 |
| 12 | 5 AND 8 AND 11 |
| **Number** | **Cochrane Library** |
| 1 | MeSH descriptor: [Flurbiprofen] explode all trees |
| 2 | ("flurbiprofen axetil" or "flurbiprofen" or "LFP83"):ti,ab,kw (Word variations have been searched) |
| 3 | 1 OR 2 |
| 4 | ("parecoxib" or "parecoxib sodium" or "Dynastat"):ti,ab,kw (Word variations have been searched) |
| 5 | MeSH descriptor: [Child, Preschool] explode all trees |
| 6 | MeSH descriptor: [Child] explode all trees |
| 7 | MeSH descriptor: [Adolescent] explode all trees |
| 8 | (Preschool Child or Children, Preschool or Preschool Children or Children or Adolescents or Adolescence or Teens or Teen or Teenagers or Teenager or Youth or Youths or Adolescents, Female or Adolescent, Female or Female Adolescent or Female Adolescents or Adolescents, Male or Adolescent, Male or Male Adolescent or Male Adolescents):ti,ab,kw (Word variations have been searched) |
| 9 | 5 OR 6 OR 7 OR 8 |
| 10 | MeSH descriptor: [Postoperative Period] explode all trees |
| 11 | MeSH descriptor: [Perioperative Period] explode all trees |
| 12 | MeSH descriptor: [Intraoperative Period] explode all trees |
| 13 | MeSH descriptor: [Preoperative Period] explode all trees |
| 14 | MeSH descriptor: [Surgical Procedures, Operative] explode all trees |
| 15 | MeSH descriptor: [General Surgery] explode all trees |
| 16 | 10 OR 11 OR 12 OR 13 OR 14 OR 15 |
| 17 | (Period, Postoperative or Periods, Postoperative or Postoperative Periods or Period, Perioperative or Periods, Perioperative or Perioperative Periods or Intraoperative Periods or Period, Intraoperative or Periods, Intraoperative or Period, Preoperative or Operative Procedures or Operative Procedure or Procedure, Operative or Procedures, Operative or Surgical Procedure, Operative or Operative Surgical Procedures or Procedure, Operative Surgical or Procedures, Operative Surgical or Surgical Procedures or Procedure, Surgical or Procedures, Surgical or Surgical Procedure or Operative Surgical Procedure or Surgery, Ghost or Ghost Surgery or Surgery, General or Surgery):ti,ab,kw (Word variations have been searched) |
| 18 | 16 OR 17 |
| 19 | 3 OR 4 |
| 20 | 18 AND 19 AND 9 |

Supplemental Table 2: The characteristics of included studies.

| No. | First author and year | Surgery Type | Age (years) | Weight (kg) | Gender (male/female) | Sample Size | Treatment (n) | Outcomes |
| --- | --- | --- | --- | --- | --- | --- | --- | --- |
| 1 | Zhu 2020 | Tonsillectomy | 3~8 | 13~38 | / | 44 | Flurbiprofen axetil 1mg/kg (22); Saline 1mg/kg (22) | 1,2 |
| 2 | Xiu 2019 | Laparoscopic hernia surgery | 2~6 | / | 45/45 | 60 | Parecoxib 1mg/kg (30); Saline 2ml (30) | 3,4,5,7,9,10,11,12 |
| 3 | Zhang 2017 | Laparoscopic surgery | 2~7 | 11~27 | 157/3 | 160 | Flurbiprofen axetil 1mg/kg (40); Tramadol 1mg/kg (40); Fentanyl 2ug/kg (40); Saline 3ml (40) | 1,2,3 |
| 4 | Li 2017 | Circumcision | 4~11 | / | / | 60 | Flurbiprofen axetil 1mg/kg (20); Flurbiprofen axetil 1.5mg/kg (20); Blank control (20) | 4,5,6,8,9 |
| 5 | He 2017 | Laparoscopic appendectomy | 3~12 | / | 30/30 | 60 | Parecoxib 1mg/kg (30); Saline 1mg/kg (30) | 10,11,12 |
| 6 | Zeng 2017 | Tonsillectomy | 5~9 | 14~30 | 28/32 | 60 | Parecoxib 1mg/kg (30); Saline 1mg/kg (30) | 3,6,7,8,10,11 |
| 7 | Li 2016 | Tonsillectomy | 3~7 | / | 30/30 | 60 | Parecoxib 1mg/kg (30); Saline 1mg/kg (30) | 10,11 |
| 8 | Tao 2016 | Plastic surgery | 3~6 | 14~30 | 65/55 | 120 | Parecoxib 0.5mg/kg (30); Parecoxib 0.75mg/kg (30); Parecoxib 1mg/kg (30); Saline (30) | 2,10,12 |
| 9 | Li 2016 | Tonsillectomy | 4~11 | 16~40 | 49/41 | 90 | Flurbiprofen axetil 1mg/kg (30); Tramadol 2mg/kg (30); Fentanyl 1ug/kg (30) | 1,10 |
| 10 | Zhang 2015 | Laparoscopic surgery | 6months~7 | 11~27 | / | 160 | Flurbiprofen axetil 1mg/kg (40); Tramadol 1mg/kg (40); Fentanyl 2ug/kg (40); Saline (40) | 1,5,7,9 |
| 11 | Yang 2015 | Tonsillectomy and adenoidectomy | 2~8 | 10~38 | 27/23 | 50 | Flurbiprofen axetil 1mg/kg (25); Saline 1mg/kg (25) | 1,2,3,4,6,10,11 |
| 12 | Zhang 2014 | Plastic surgery | / | / | / | 60 | Flurbiprofen axetil 1mg/kg (20); Tramadol 1.5mg/kg (20); Saline 2ml (20) | 1,3,5,7,8,9,10,11,12 |
| 13 | Zhang 2014 | Laparoscopic hernia surgery | 1~10 | / | / | 60 | Parecoxib 1mg/kg (20); Tramadol 1.5mg/kg (20); Saline 2ml (20) | 1,4,5,7,8,10,11 |
| 14 | Yang 2014 | Tonsillectomy and adenoidectomy | 4~9 | 15~36 | 27/18 | 30 | Flurbiprofen axetil 1mg/kg (15); Saline (15) | 4,6,8,9,10,11,12 |
| 15 | Wang 2014 | Tonsillectomy | 4~12 | / | / | 40 | Parecoxib 0.8mg/kg (20); Saline (20) | 2,3,4,5,7,8,9,10,12 |
| 16 | Sun 2014 | Tonsillectomy | 4~10 | 16~32 | 96/24 | 90 | Parecoxib 0.5mg/kg (30); Tramadol 1mg/kg (30); Saline 5ml (30) | 3,5,8,9,10 |
| 17 | Li 2014 | Tonsillectomy | 4~12 | 15~42 | 47/43 | 60 | Flurbiprofen axetil 1mg/kg (30); Saline 5ml (30) | 3,4,5,7,8,9 |
| 18 | Li 2014 | Inguinal herniorrhaphy | 3~8 | / | 53/47 | 80 | Flurbiprofen axetil 0.5mg/kg (20); Flurbiprofen axetil 1mg/kg (20); Flurbiprofen axetil 1.5mg/kg (20); Blank control (20) | 4,5,6,7,8,10 |
| 19 | Li 2014 | Tonsillectomy and adenoidectomy | 5~8 | 18~30 | / | 60 | Parecoxib 1mg/kg (20); Flurbiprofen axetil 1mg/kg (20); Saline 2ml (20) | 3,4,5,6,8, 10,11,12 |
| 20 | Yi 2013 | Tonsillectomy | 4~12 | / | 33/27 | 40 | Flurbiprofen axetil 1mg/kg (20); Saline 2ml (20) | 10,11,12 |
| 21 | Ye 2013 | Strabismus surgery | 6~12 | / | / | 60 | Parecoxib 0.5mg/kg (30); Tramadol 1.5mg/kg (30) | 4,5,6,7,8,10,11 |
| 22 | Ma 2013 | Tonsillectomy and adenoidectomy | 4~12 | 15~32 | 48/38 | 86 | Parecoxib 1mg/kg (43); Fentanyl 1ug/kg (43) | 3,5,7,8,9,10,11 |
| 23 | Ma 2013 | Abdominal surgery | 5~8 | 15~20 | / | 40 | Flurbiprofen axetil 1.25mg/kg (20) ; Saline 2ml (20) | 4,6,8,9 |
| 24 | Ma 2013 | Inguinal herniorrhaphy | 6.5~9 | 19~24 | / | 40 | Flurbiprofen axetil 1.25mg/kg(20); Blank control (20) | 4,6,8,9 |
| 25 | Fan 2013 | Urethroplasty | 2~8 | / | / | 32 | Parecoxib 1mg/kg (16); Saline (16) | 10,11 |
| 26 | Zhang 2012 | Orthopedic surgery | 4~8 | 15~24 | 32/28 | 40 | Flurbiprofen axetil 1mg/kg (20); Saline 5ml (20) | 3,4,5,7,10,11 |
| 27 | Miao 2012 | Tonsillectomy and adenoidectomy | 5~8 | 16~28 | 26/34 | 60 | Parecoxib 1mg/kg (20); Fentanyl 1ug/kg (20); Saline 5ml (20) | 3,4,5,6,7,10,11 |
| 28 | Kong 2012 | Tonsillectomy and adenoidectomy | 4~14 | 14~50 | 50/30 | 60 | Flurbiprofen axetil 1mg/kg (20); Tramadol 2mg/kg (20); Saline 3ml (20) | 1,5,7,9,10,11,12 |
| 29 | Huang 2012 | Circumcision | 3~12 | 11~40 | / | 60 | Flurbiprofen axetil 1mg/kg (30); Blank control (30) | 2,3,4,5,7,8,9,10,11 |
| 30 | Yang 2011 | Inguinal herniorrhaphy | 2~6 | 10~25 | 34/26 | 40 | Flurbiprofen axetil 1mg/kg (20); Saline 5ml (20) | 3,4,5,7,8,9,10,11 |
| 31 | Tian 2011 | Plastic surgery | 4~10 | 11~37 | 74/16 | 60 | Flurbiprofen axetil 1mg/kg (30); Saline 3ml (30) | 3,4,5,7,8,9,10,11 |
| 32 | Peng 2011 | Tonsillectomy and adenoidectomy | 3~7 | 10~31 | / | 60 | Flurbiprofen axetil 1mg/kg (20); Flurbiprofen axetil 1.5mg/kg (20); Saline 10ml (20) | 2,3,5,7,8,9,10,11,12 |
| 33 | Peng 2011 | Adenoidectomy | 5~10 | 18~30 | 38/52 | 60 | Flurbiprofen axetil 1mg/kg (30); Saline 5ml (30) | 3,4,5,7,8,9,10,11 |
| 34 | Lin 2011 | Tonsillectomy | 6~12 | 17~39 | 48/42 | 90 | Flurbiprofen axetil 1mg/kg (30); Fentanyl 1ug/kg (30); Tramadol 2mg/kg (30) | 2,4,6,10,11,12 |
| 35 | Li 2011 | Orthopedic surgery | 3~5 | 10~25 | 34/26 | 90 | Parecoxib 1mg/kg (30); Tramadol 2mg/kg (30); Saline (30) | 4,5,6,7,10,11 |
| 36 | Li 2011 | Tonsillectomy | 4~12 | / | / | 80 | Flurbiprofen axetil 0.5mg/kg (20); Flurbiprofen axetil 1mg/kg (20); Flurbiprofen axetil 1.5mg/kg (20); Blank control (20) | 4,5,6,7,8,10,11 |
| 37 | Li 2011 | Abdominal surgery | 3~12 | / | / | 90 | Parecoxib 0.5mg/kg (30); Tramadol 2mg/kg (30); Saline (30) | 2,3,10,12 |
| 38 | Lei 2011 | Tonsillectomy and adenoidectomy | 5~9 | 16~32 | 22/18 | 40 | Flurbiprofen axetil 1mg/kg (20); Saline 10ml (20) | 3,4,5,7,8,9,10,11 |
| 39 | He 2011 | Strabismus surgery | 3~7 | 12~26 | / | 60 | Flurbiprofen axetil 1mg/kg (30); Saline 5ml (30) | 3,4,5,6,7,8,9,10,11,12 |
| 40 | Yuan 2010 | Orthopedic surgery | 5~14 | / | 52/38 | 60 | Parecoxib 0.5mg/kg (30); Tramadol 1mg/kg (30) | 10,11 |
| 41 | Wei 2010 | Tonsillectomy | 5~10 | 16~31 | / | 50 | Flurbiprofen axetil 0.4mg/kg (10); Flurbiprofen axetil 0.6mg/kg (10); Flurbiprofen axetil 0.8mg/kg (10); Flurbiprofen axetil 1mg/kg (10); Fentanyl 1ug/kg (10) | 3,4,5,7,8,9 |
| 42 | Lu 2010 | Circumcision | / | / | / | 60 | Flurbiprofen axetil 1mg/kg (30); Saline 10ml (30) | 3,4,5,7,8,9,10,11 |
| 43 | Chen 2010 | Tonsillectomy and adenoidectomy | 2.5~8 | 11~40 | 39/21 | 60 | Flurbiprofen axetil 1mg/kg (20); Tramadol 1mg/kg (20); Saline (20) | 3,5,6,8,10,11,12 |
| 44 | Mo 2009 | Inguinal herniorrhaphy | 3~5 |  | / | 120 | Flurbiprofen axetil 1mg/kg (60); Saline 0.1mg/kg (60) | 3,4,5,7,9,10,11 |
| 45 | Long 2008 | Inguinal herniorrhaphy | 7~12 | 18~40 | / | 60 | Flurbiprofen axetil 1mg/kg (30); Saline (30) | 4,6,8,9,10,11 |
| 46 | Zeng 2008 | Tonsillectomy | 3~12 | 15~40 | 32/28 | 40 | Flurbiprofen axetil 1mg/kg (20); Saline 5ml (20) | 3,4,5,7,8,9,10,11 |
| 47 | Zeng 2008 | Inguinal herniorrhaphy | 1~12 | 8~40 | 29/31 | 40 | Flurbiprofen axetil 1mg/kg (20); Saline 5ml (20) | 3,4,5,7,8,9,10,11 |
| 48 | Sun 2007 | Tonsillectomy and adenoidectomy | 4~9 | 13~26.5 | 23/9 | 32 | Flurbiprofen axetil 1mg/kg (16); Lipid emulsion 0.1mg/kg (16) | 1,2,3,4,5,6,7,10 |
| 49 | Mikawa 1997 | Strabismus surgery | 2~11 | / | / | 90 | Flurbiprofen axetil 0.5mg/kg (30); Flurbiprofen axetil 1mg/kg (30); Saline (30) | 10,11 |

1. Pain scores at postoperative 0 h. 2. Pain scores at postoperative 0.5 h. 3. Pain scores at postoperative 1 h. 4. Pain scores at postoperative 2 h. 5. Pain scores at postoperative 4 h. 6. Pain scores at postoperative 6 h. 7. Pain scores at postoperative 8 h. 8. Pain scores at postoperative 12 h. 9. Pain scores at postoperative 24 h. 10. Total adverse events. 11. Postoperative nausea and vomiting. 12. Agitaion.

Supplemental Table 3: Ranking results of all outcomes.

| Outcomes | Rank | Fentanyl | Flurbiprofen | Parecoxib | Placebo | Tramadol |
| --- | --- | --- | --- | --- | --- | --- |
| Pain scores at postoperative 0h | V1 | 0.11824 | 0.001735 | 0.035885 | 0.842225 | 0.001915 |
|  | V2 | 0.576685 | 0.113835 | 0.09923 | 0.145215 | 0.065035 |
|  | V3 | 0.18395 | 0.40601 | 0.12271 | 0.0115 | 0.27583 |
|  | V4 | 0.084685 | 0.332215 | 0.12181 | 0.000945 | 0.460345 |
|  | V5 | 0.03644 | 0.146205 | 0.620365 | 0.000115 | 0.196875 |
| Pain scores at postoperative 0.5h | V1 | 0.01871 | 0.003405 | 0.00155 | 0.97313 | 0.003205 |
|  | V2 | 0.247775 | 0.56361 | 0.062975 | 0.02402 | 0.10162 |
|  | V3 | 0.324825 | 0.29709 | 0.127405 | 0.00254 | 0.24814 |
|  | V4 | 0.24437 | 0.110945 | 0.21842 | 0.000295 | 0.42597 |
|  | V5 | 0.16432 | 0.02495 | 0.58965 | 0.000015 | 0.221065 |
| Pain scores at postoperative 1h | V1 | 0.0001 | 0 | 0.000005 | 0.99984 | 0.000055 |
|  | V2 | 0.21083 | 0.179235 | 0.06692 | 0.00016 | 0.542855 |
|  | V3 | 0.213555 | 0.38251 | 0.16376 | 0 | 0.240175 |
|  | V4 | 0.254 | 0.296645 | 0.311785 | 0 | 0.13757 |
|  | V5 | 0.321515 | 0.14161 | 0.45753 | 0 | 0.079345 |
| Pain scores at postoperative 2h | V1 | 0.00001 | 0 | 0 | 0.99997 | 0.00002 |
|  | V2 | 0.167425 | 0.04534 | 0.150825 | 0.00003 | 0.63638 |
|  | V3 | 0.22345 | 0.16078 | 0.397685 | 0 | 0.218085 |
|  | V4 | 0.240905 | 0.37204 | 0.291525 | 0 | 0.09553 |
|  | V5 | 0.36821 | 0.42184 | 0.159965 | 0 | 0.049985 |
| Pain scores at postoperative 4h | V1 | 0.007515 | 0 | 0 | 0.99245 | 0.000035 |
|  | V2 | 0.69441 | 0.005325 | 0.018285 | 0.00755 | 0.27443 |
|  | V3 | 0.22007 | 0.058005 | 0.134865 | 0 | 0.58706 |
|  | V4 | 0.055425 | 0.336465 | 0.493295 | 0 | 0.114815 |
|  | V5 | 0.02258 | 0.600205 | 0.353555 | 0 | 0.02366 |
| Pain scores at postoperative 6h | V1 | 0.001555 | 0 | 0 | 0.998195 | 0.00025 |
|  | V2 | 0.33295 | 0.32864 | 0.02612 | 0.00177 | 0.31052 |
|  | V3 | 0.22464 | 0.36955 | 0.07932 | 0.000035 | 0.326455 |
|  | V4 | 0.255625 | 0.24295 | 0.23171 | 0 | 0.269715 |
|  | V5 | 0.18523 | 0.05886 | 0.66285 | 0 | 0.09306 |
| Pain scores at postoperative 8h | V1 | 0.0005 | 0 | 0.000005 | 0.997825 | 0.00167 |
|  | V2 | 0.167255 | 0.0109 | 0.010735 | 0.002155 | 0.808955 |
|  | V3 | 0.493625 | 0.197445 | 0.14516 | 0.00002 | 0.16375 |
|  | V4 | 0.203385 | 0.42605 | 0.348815 | 0 | 0.02175 |
|  | V5 | 0.135235 | 0.365605 | 0.495285 | 0 | 0.003875 |
| Pain scores at postoperative 12h | V1 | 0.021785 | 0 | 0.000165 | 0.977635 | 0.000415 |
|  | V2 | 0.38295 | 0.023385 | 0.26617 | 0.022255 | 0.30524 |
|  | V3 | 0.171585 | 0.09673 | 0.4121 | 0.000105 | 0.31948 |
|  | V4 | 0.186335 | 0.297435 | 0.24528 | 0.000005 | 0.270945 |
|  | V5 | 0.237345 | 0.58245 | 0.076285 | 0 | 0.10392 |
| Pain scores at postoperative 24h | V1 | 0.07468 | 0.00001 | 0.021225 | 0.78607 | 0.118015 |
|  | V2 | 0.2065 | 0.041885 | 0.10341 | 0.18298 | 0.465225 |
|  | V3 | 0.287475 | 0.219635 | 0.214255 | 0.02844 | 0.250195 |
|  | V4 | 0.229855 | 0.376105 | 0.27316 | 0.002505 | 0.118375 |
|  | V5 | 0.20149 | 0.362365 | 0.38795 | 0.000005 | 0.04819 |
| Incidence of total adverse events | V1 | 0.2244 | 0.00015 | 0 | 0.2693 | 0.50615 |
|  | V2 | 0.18285 | 0.0042 | 0 | 0.4636 | 0.34935 |
|  | V3 | 0.4499 | 0.13525 | 0.0088 | 0.26605 | 0.14 |
|  | V4 | 0.1163 | 0.74175 | 0.1364 | 0.00105 | 0.0045 |
|  | V5 | 0.02655 | 0.11865 | 0.8548 | 0 | 0 |
| Incidence of postoperative nausea and vomiting | V1 | 0.2935 | 0.00045 | 0 | 0.0009 | 0.70515 |
|  | V2 | 0.54095 | 0.027 | 0.0001 | 0.1407 | 0.29125 |
|  | V3 | 0.08005 | 0.193 | 0.0011 | 0.7228 | 0.00305 |
|  | V4 | 0.08355 | 0.7596 | 0.0208 | 0.1355 | 0.00055 |
|  | V5 | 0.00195 | 0.01995 | 0.978 | 0.0001 | 0 |
| Incidence of agitaion after surgery | V1 | 0.09525 | 0.00005 | 0.00065 | 0.8833 | 0.02075 |
|  | V2 | 0.1975 | 0.059 | 0.07895 | 0.10925 | 0.5553 |
|  | V3 | 0.1787 | 0.2656 | 0.228 | 0.00735 | 0.32035 |
|  | V4 | 0.13525 | 0.42205 | 0.3583 | 0.0001 | 0.0843 |
|  | V5 | 0.3933 | 0.2533 | 0.3341 | 0 | 0.0193 |

Supplemental Figure 1: Results of Node-splitting analysis.

A, pain scores at postoperative 0 h. B, pain scores at postoperative 0.5 h. C, pain scores at postoperative 1 h. D, pain scores at postoperative 2 h. E, pain scores at postoperative 4 h. F, pain scores at postoperative 6 h. G, pain scores at postoperative 8 h. H, pain scores at postoperative 12 h. I, pain scores at postoperative 24 h. J, incidence of total adverse events. K, incidence of postoperative nausea and vomiting. L, incidence of agitaion after surgery.


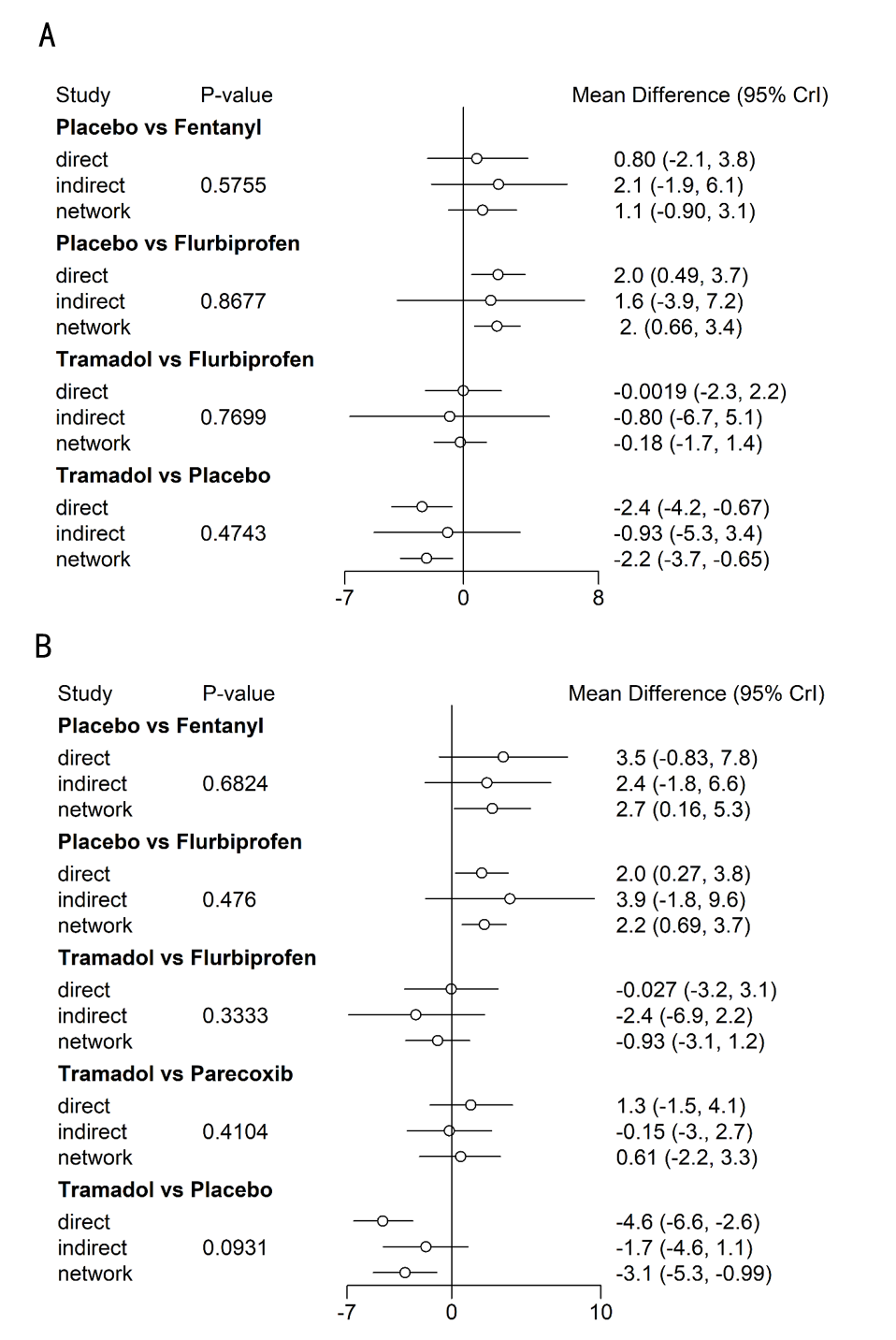

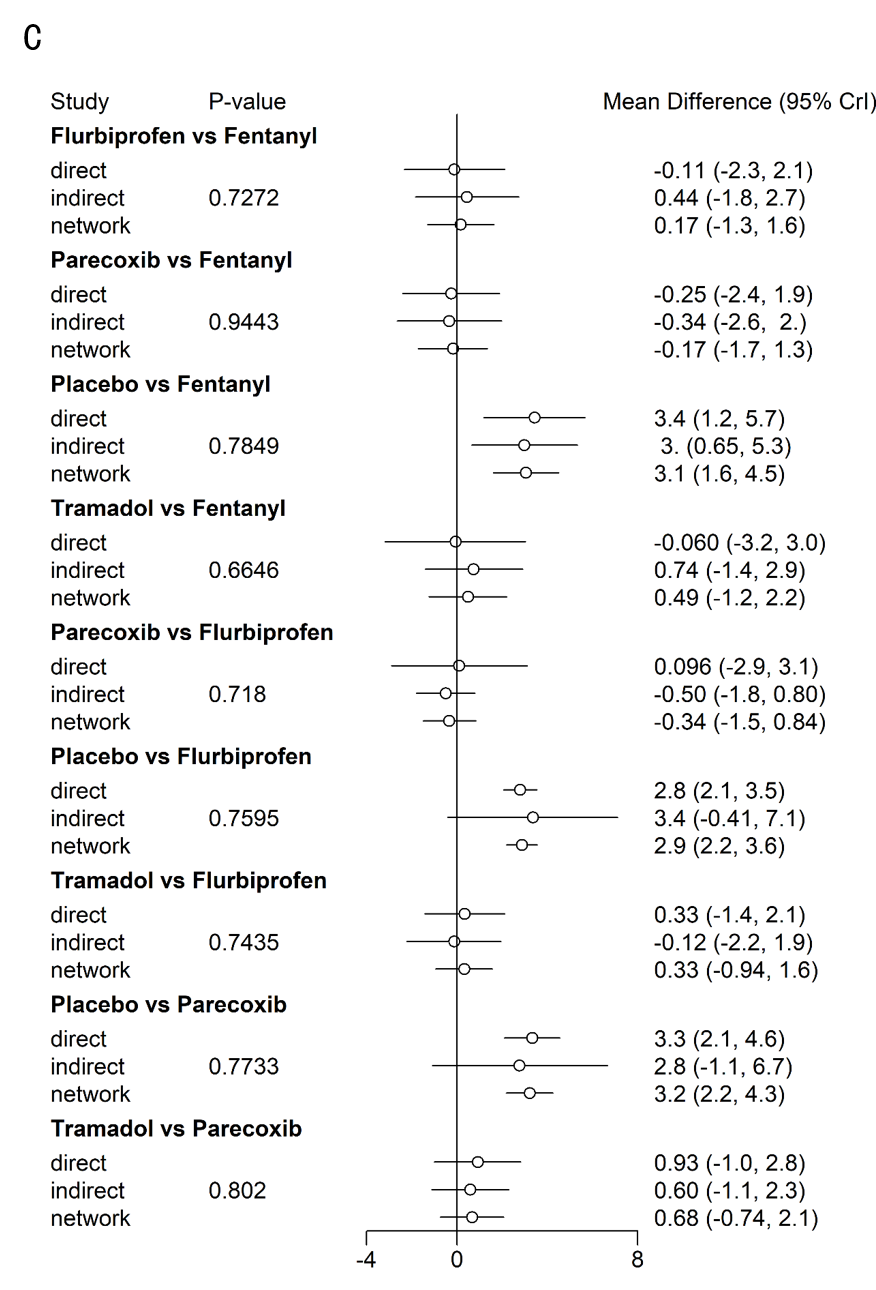


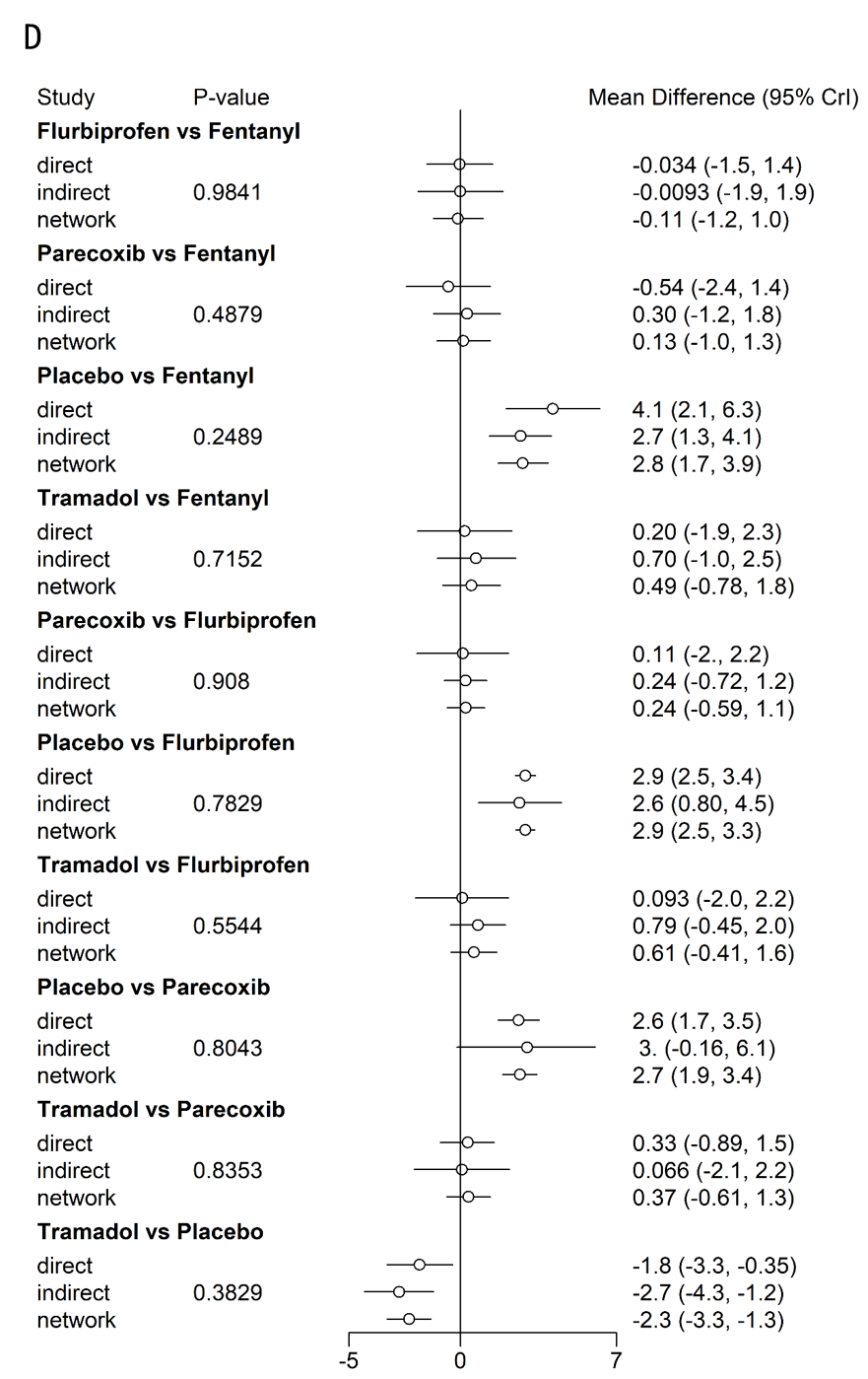

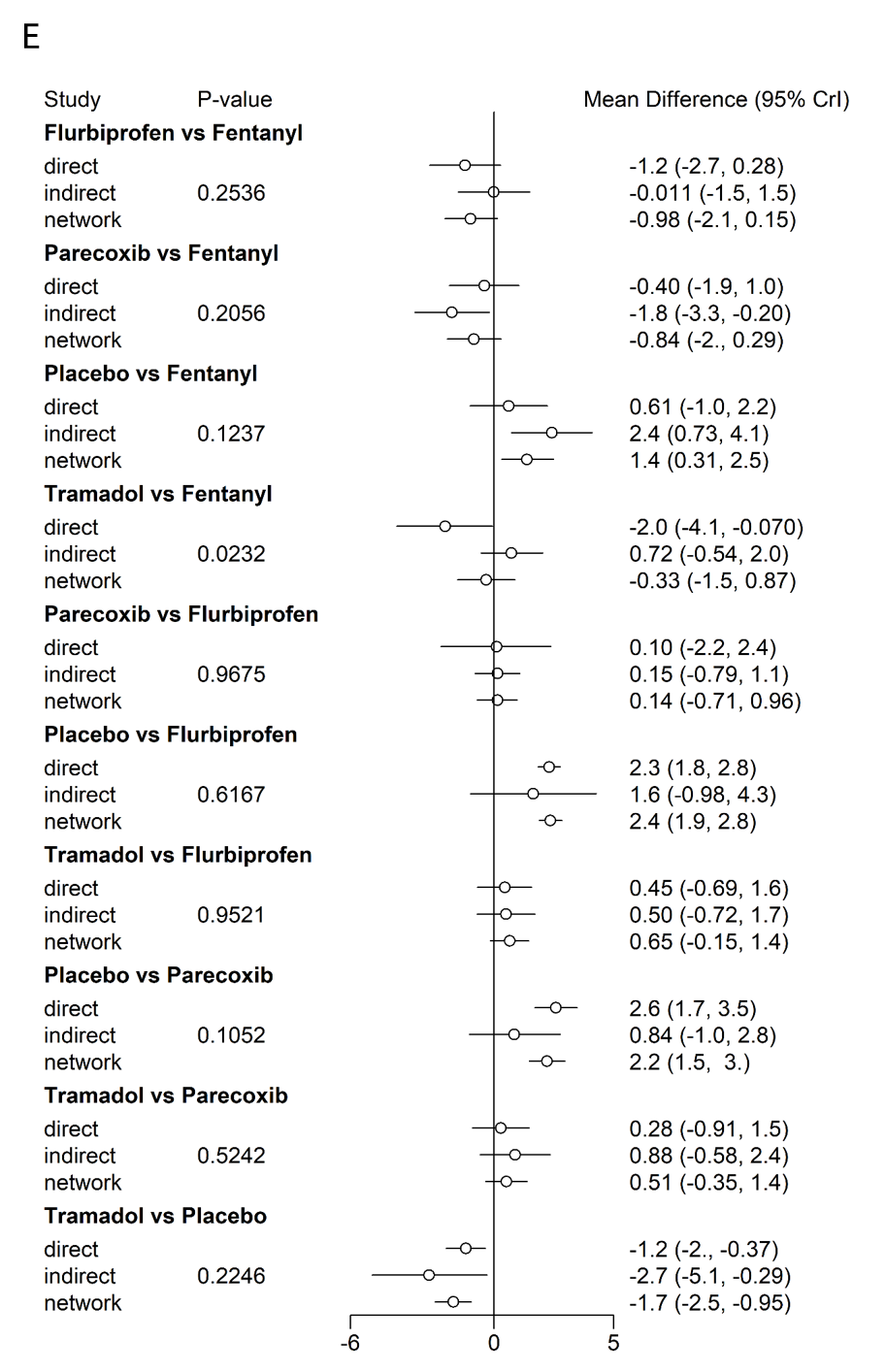


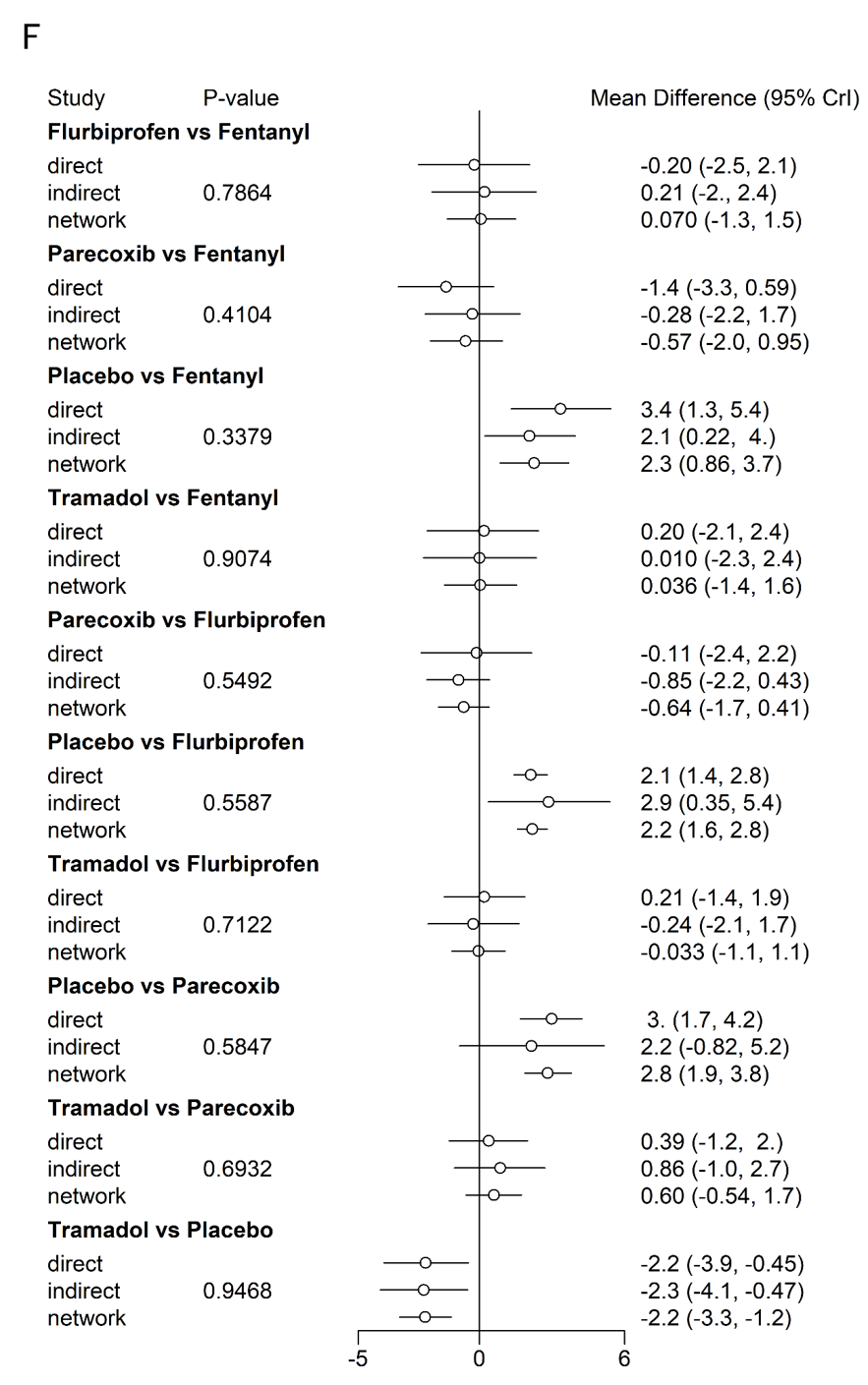

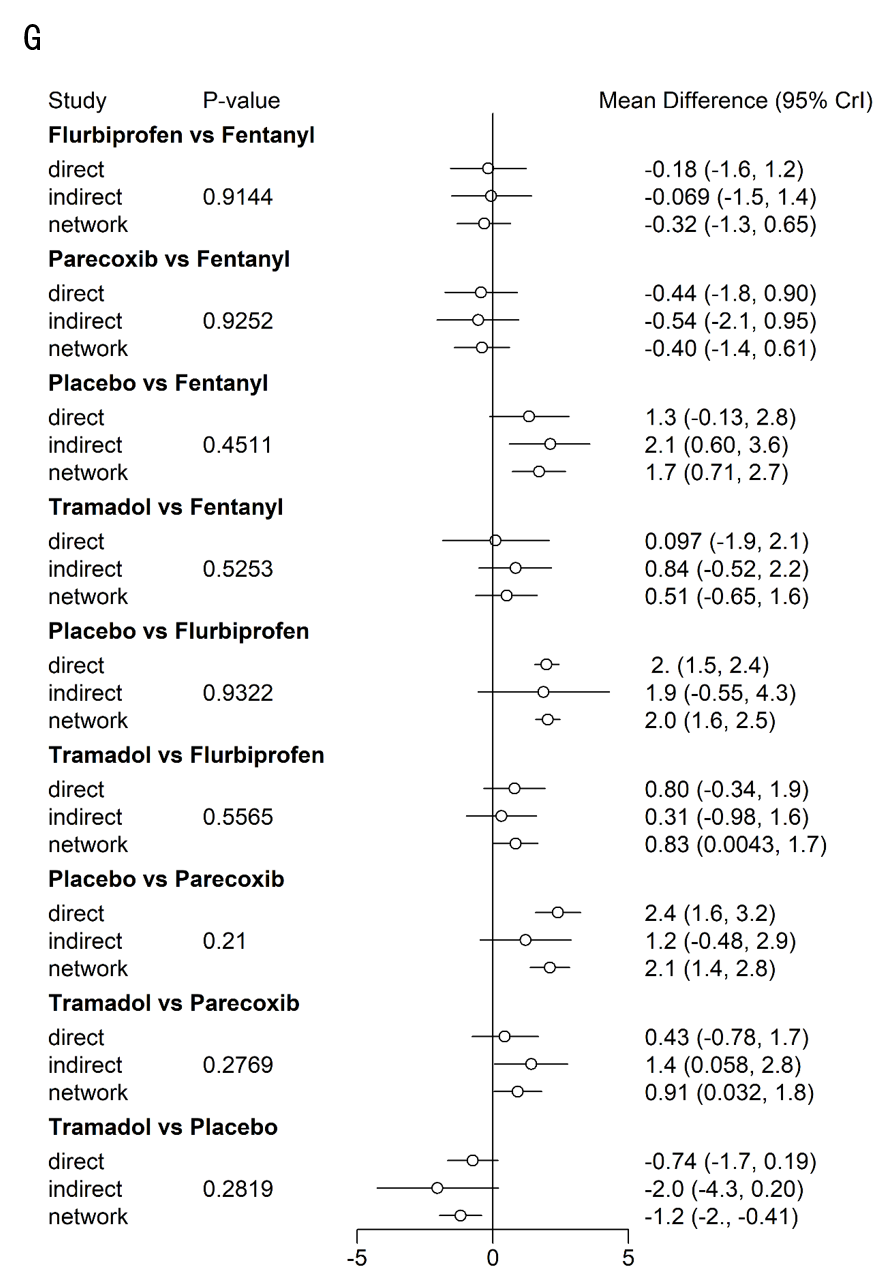


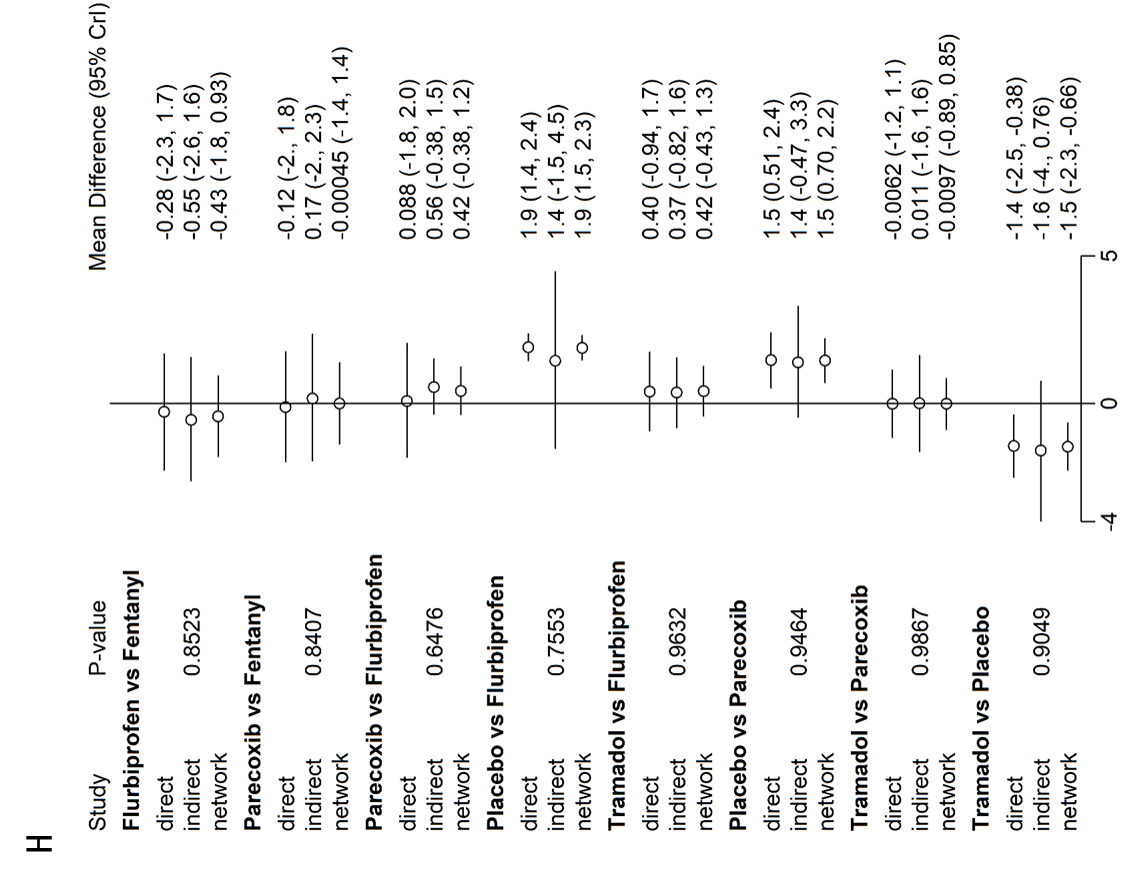

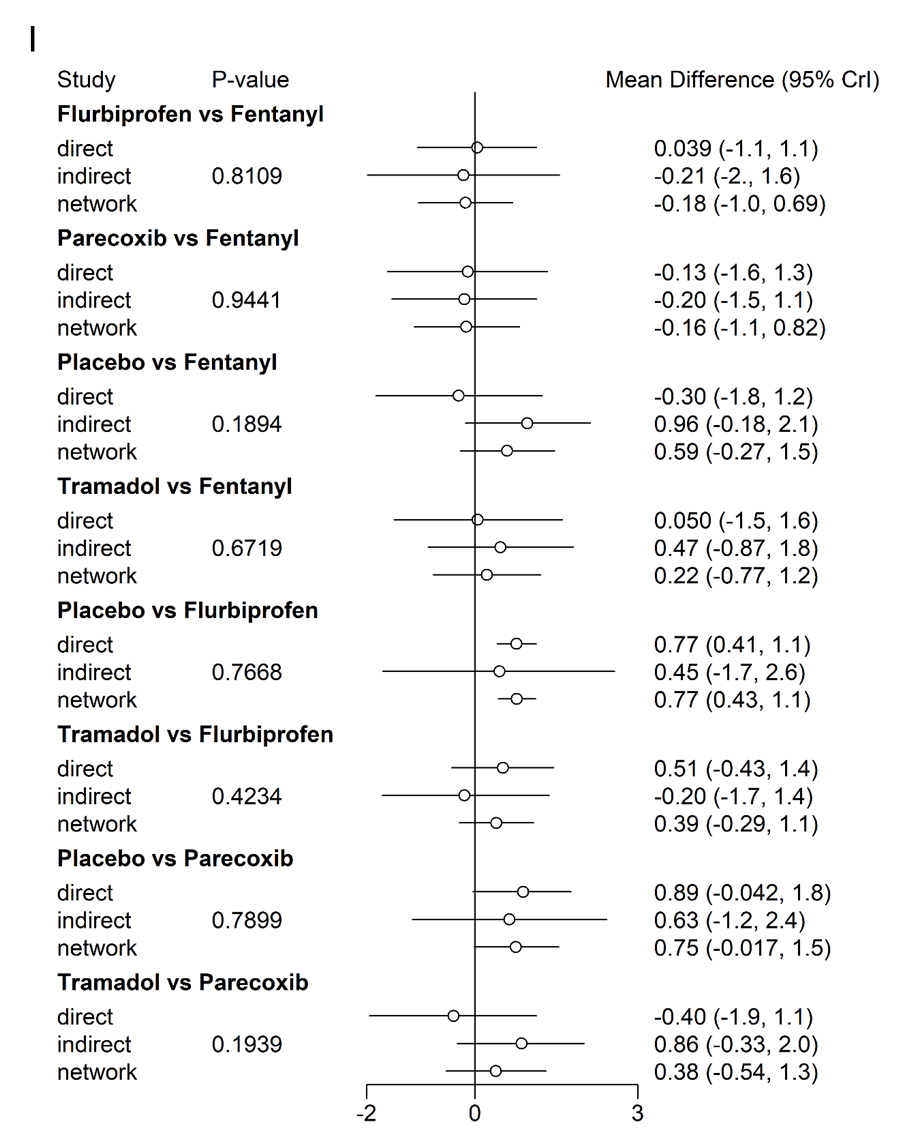


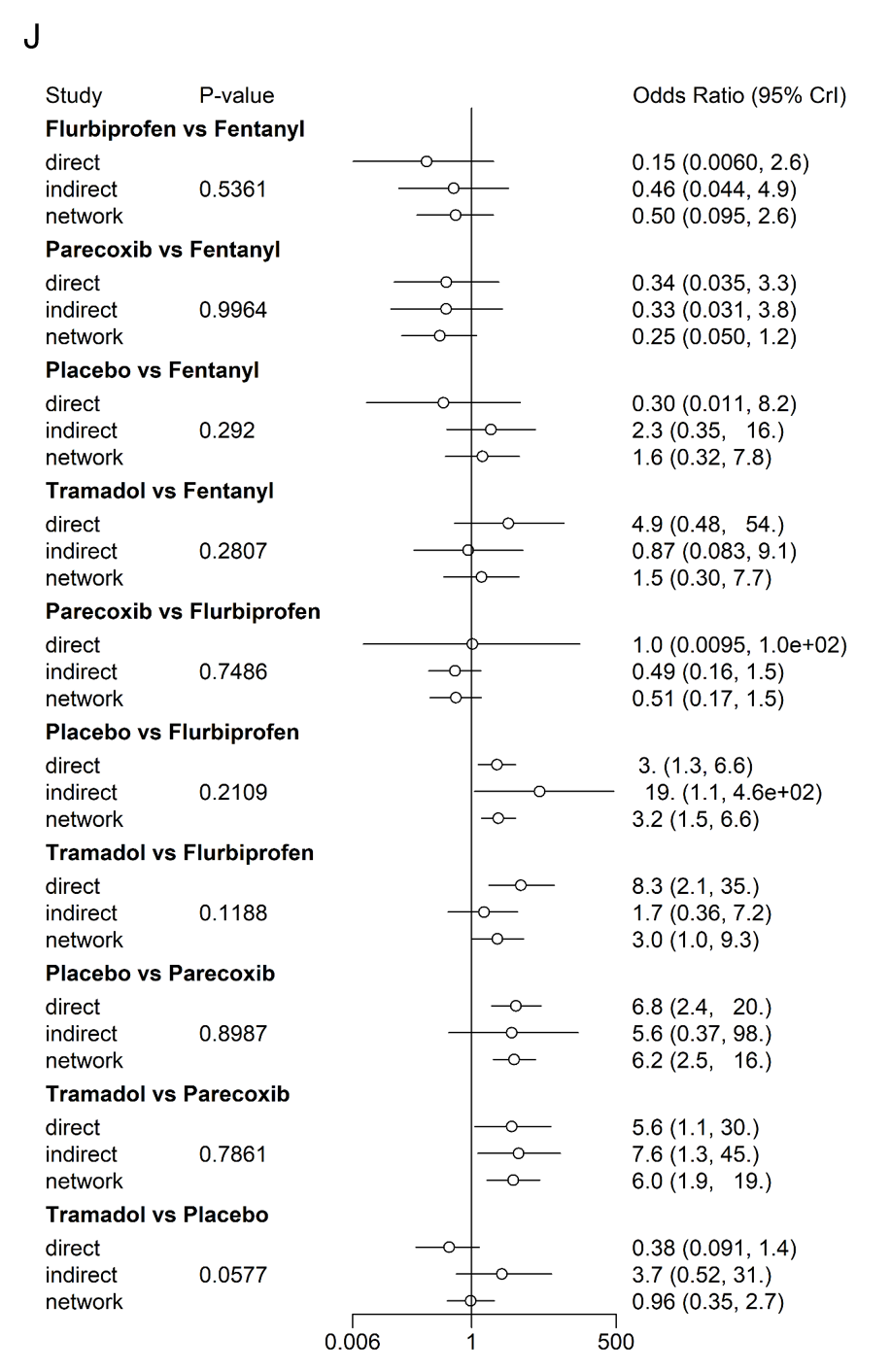

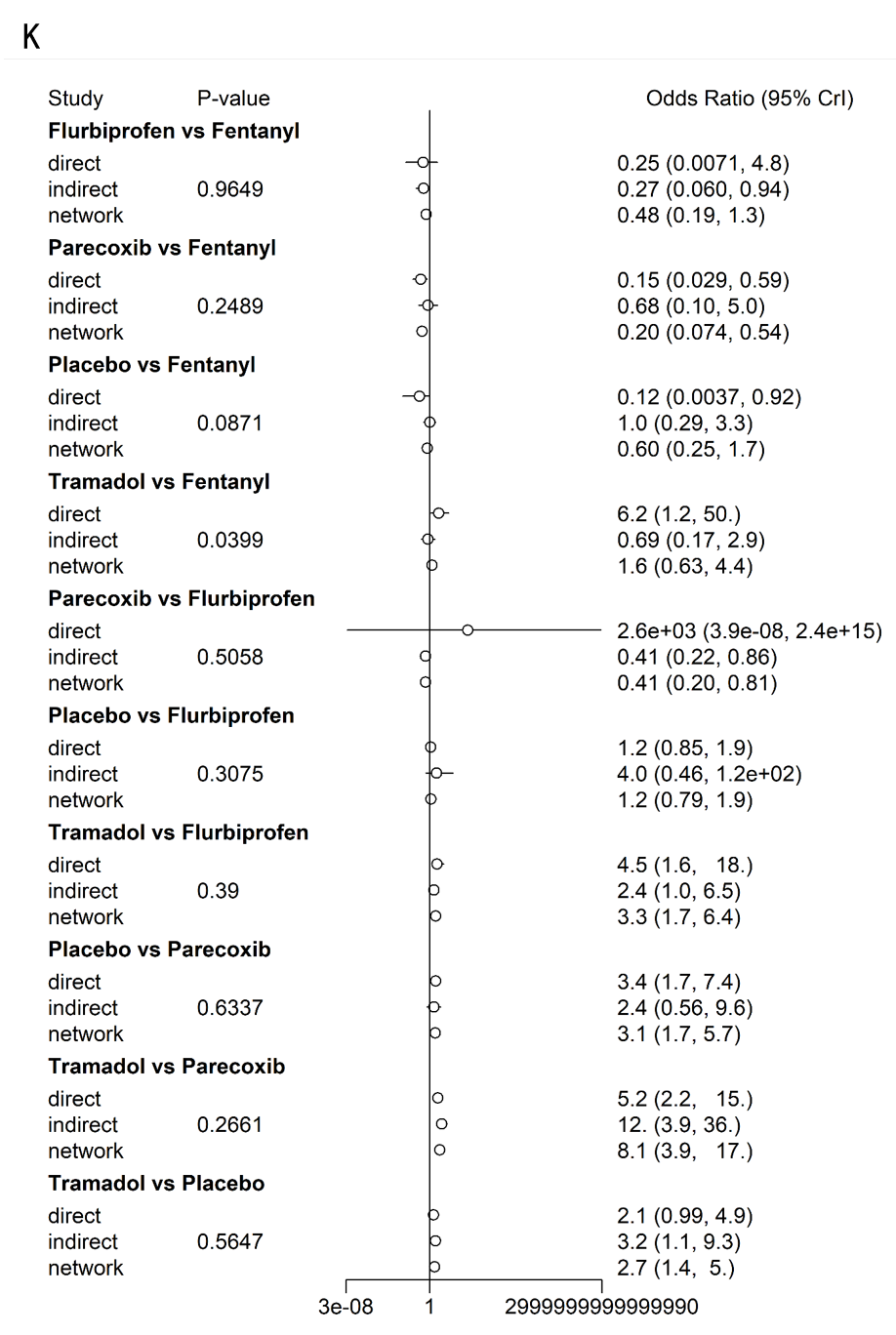


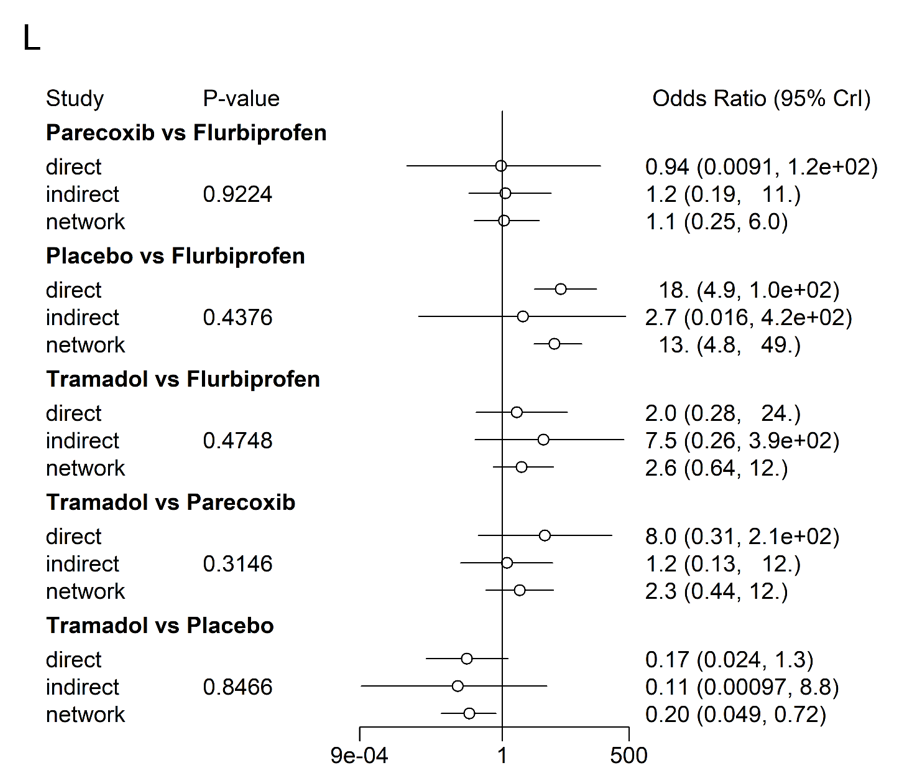


Supplemental Figure 2: Results of convergence analysis.

A, pain scores at postoperative 0 h. B, pain scores at postoperative 0.5 h. C, pain scores at postoperative 1 h. D, pain scores at postoperative 2 h. E, pain scores at postoperative 4 h. F, pain scores at postoperative 6 h. G, pain scores at postoperative 8 h. H, pain scores at postoperative 12 h. I, pain scores at postoperative 24 h. J, incidence of total adverse events. K, incidence of postoperative nausea and vomiting. L, incidence of agitaion after surgery.


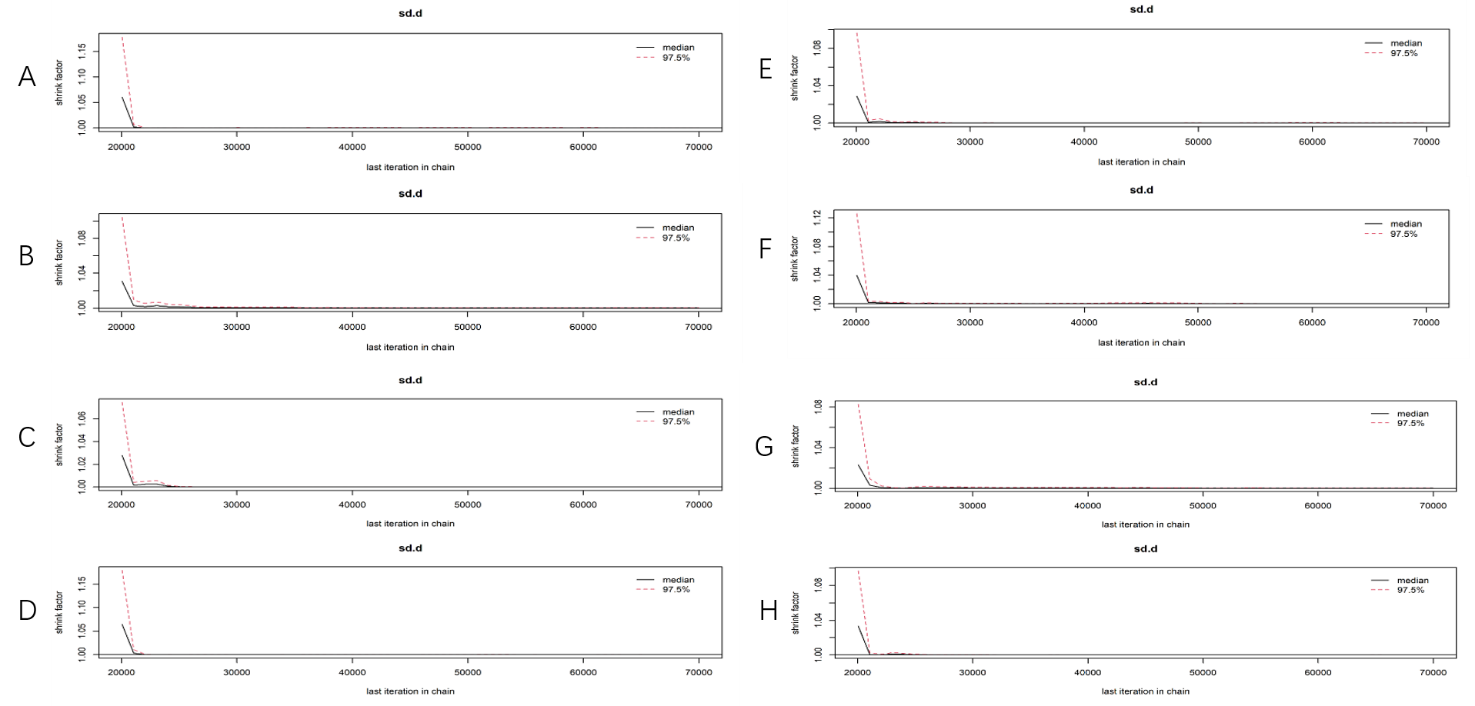

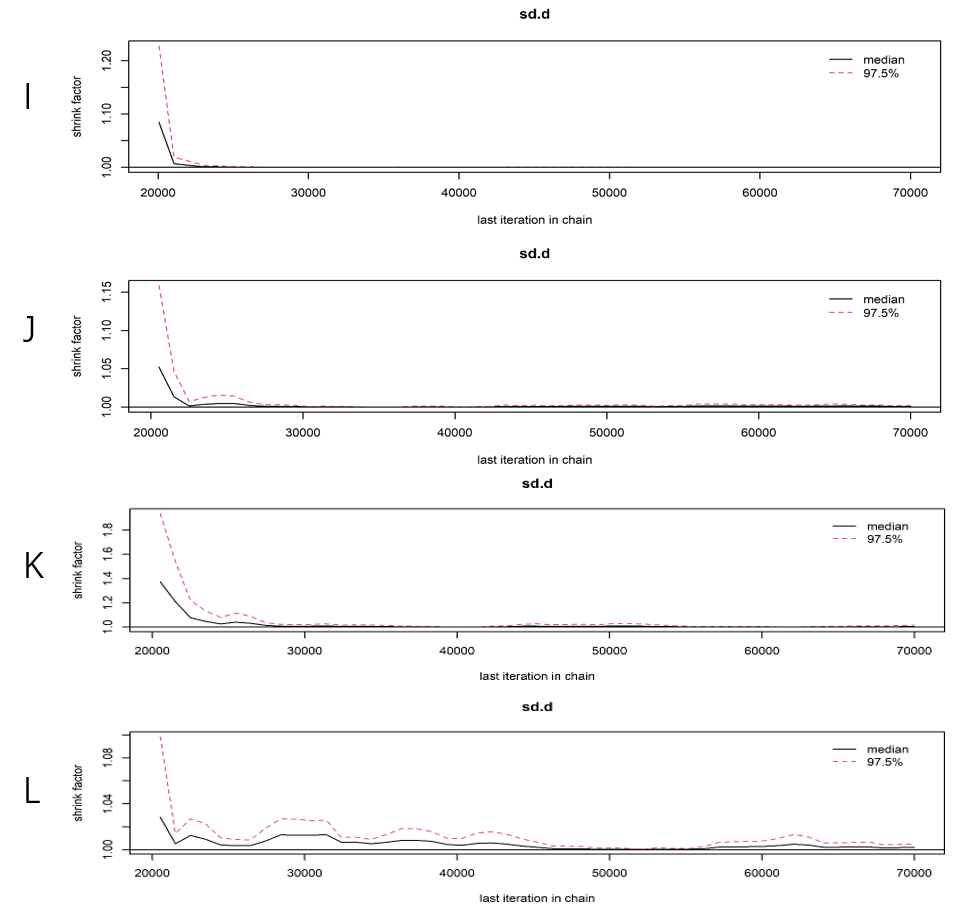


Supplemental Figure 3: The pairwise comparisons of heterogeneity.

A, pain scores at postoperative 0 h. B, pain scores at postoperative 0.5 h. C, pain scores at postoperative 1 h. D, pain scores at postoperative 2 h. E, pain scores at postoperative 4 h. F, pain scores at postoperative 6 h. G, pain scores at postoperative 8 h. H, pain scores at postoperative 12 h. I, pain scores at postoperative 24 h. J, incidence of total adverse events. K, incidence of postoperative nausea and vomiting. L, incidence of agitaion after surgery.


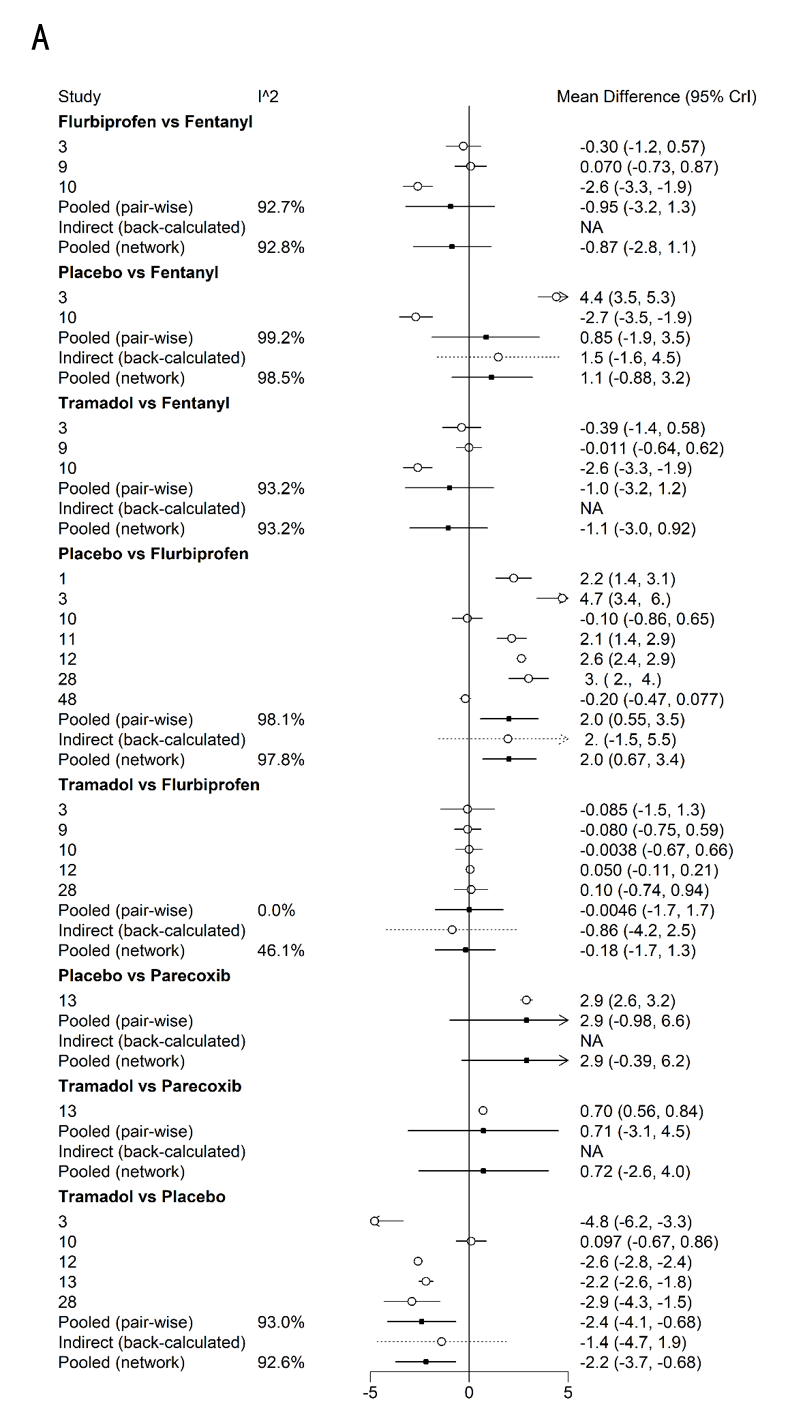

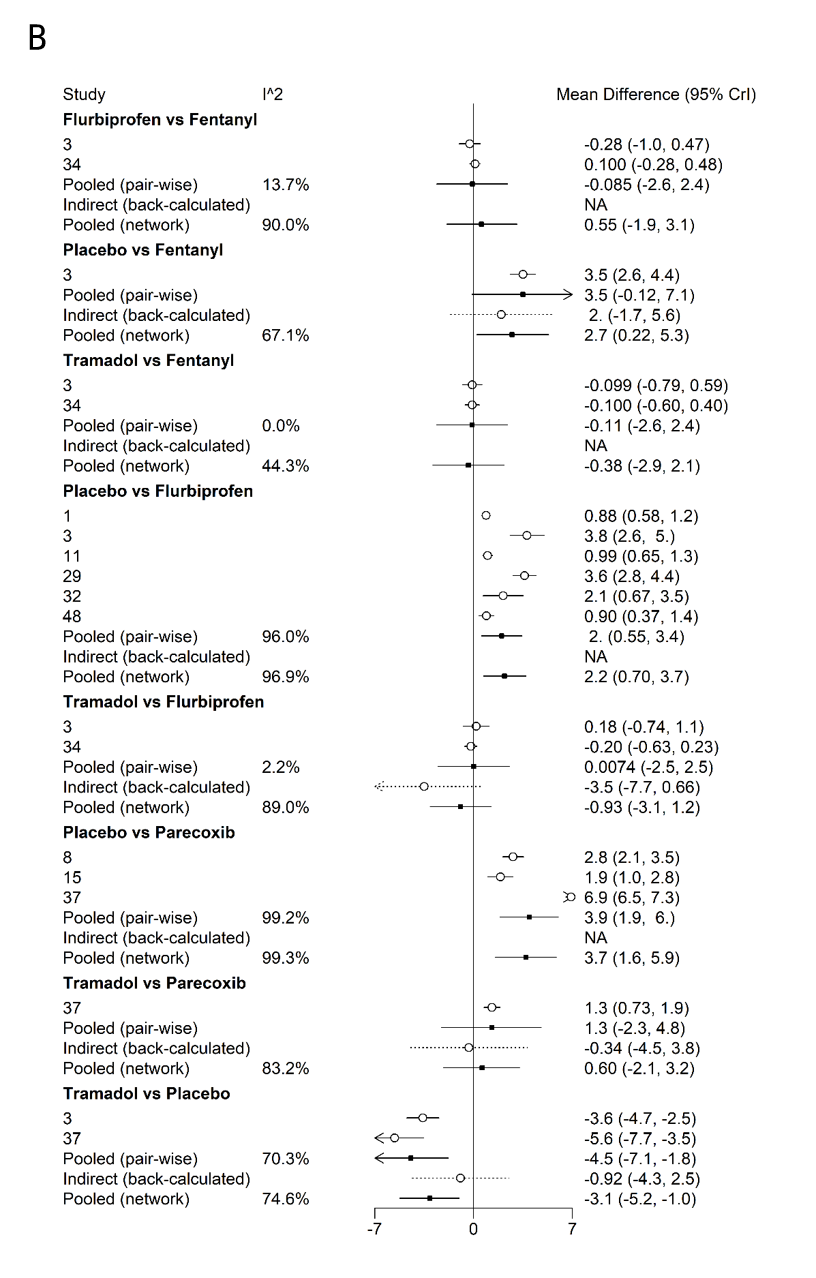


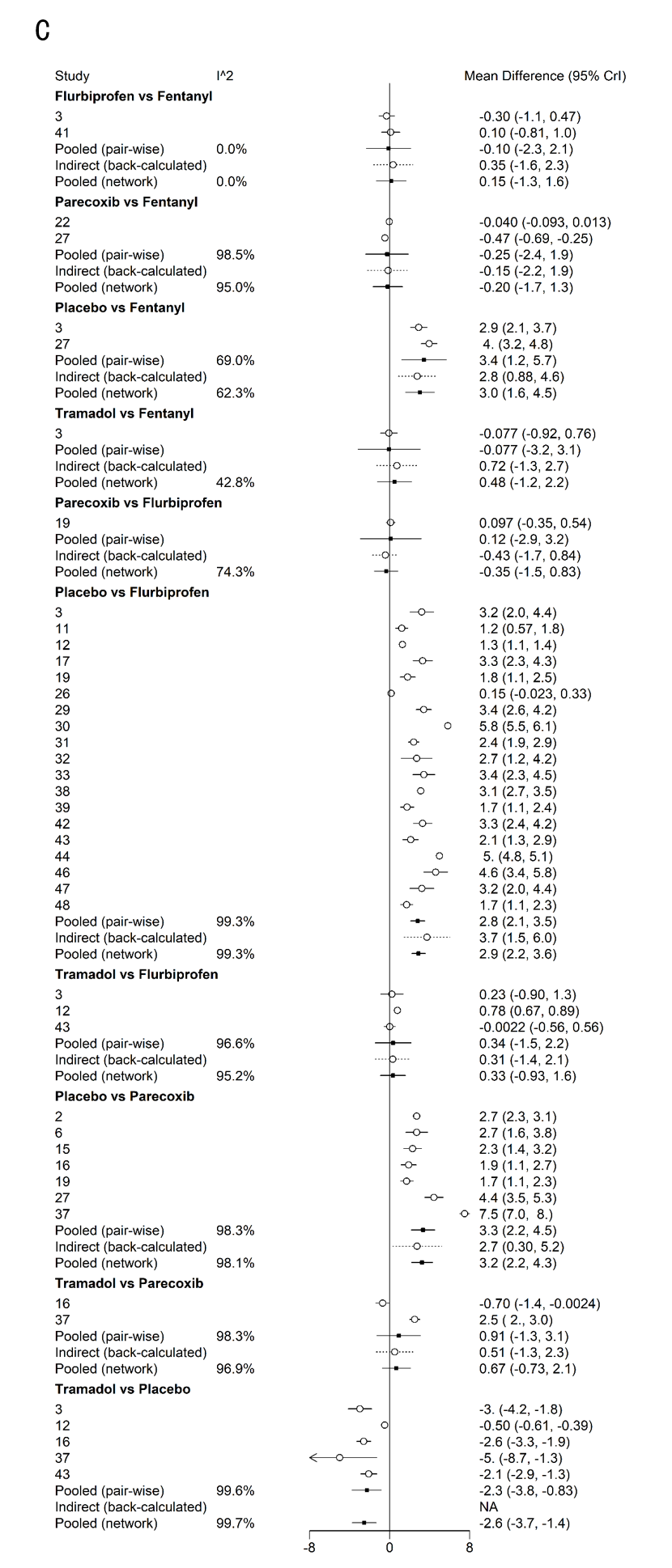

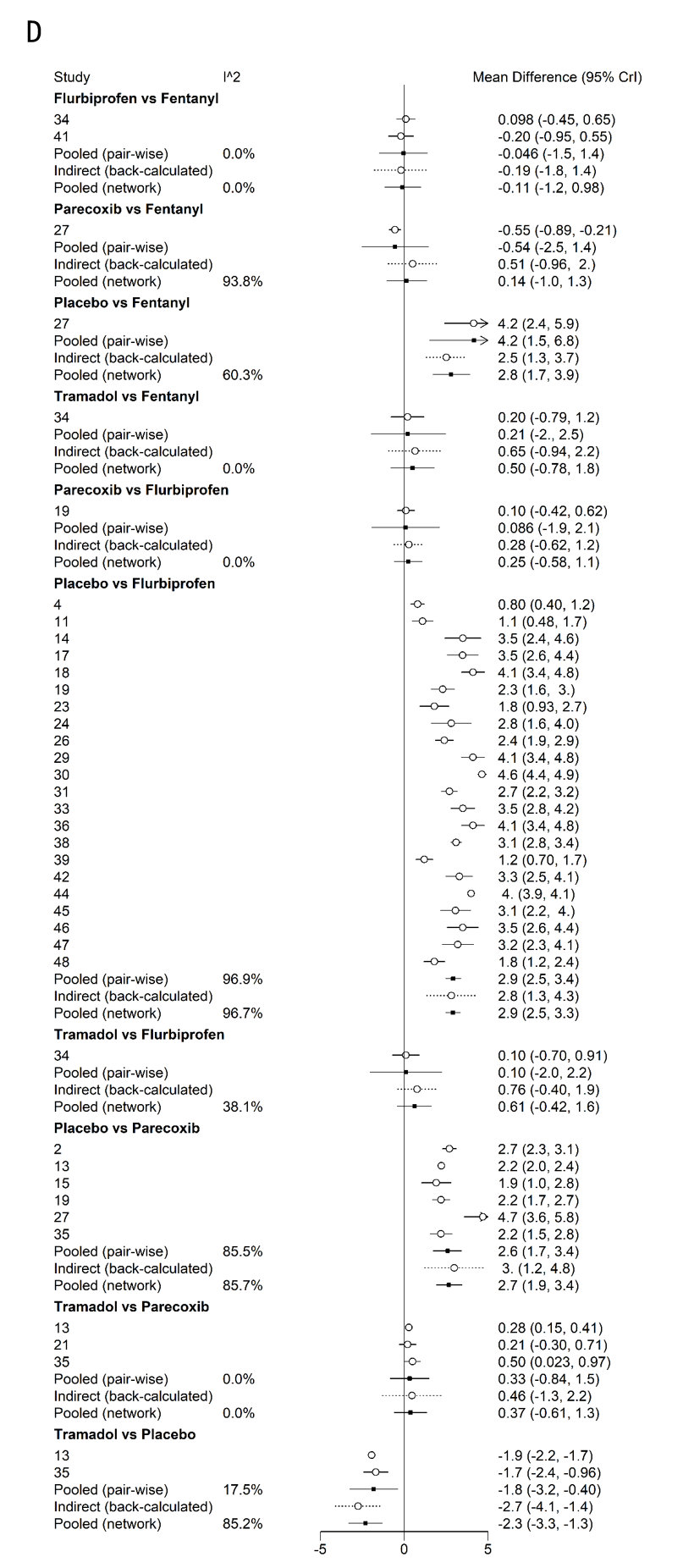


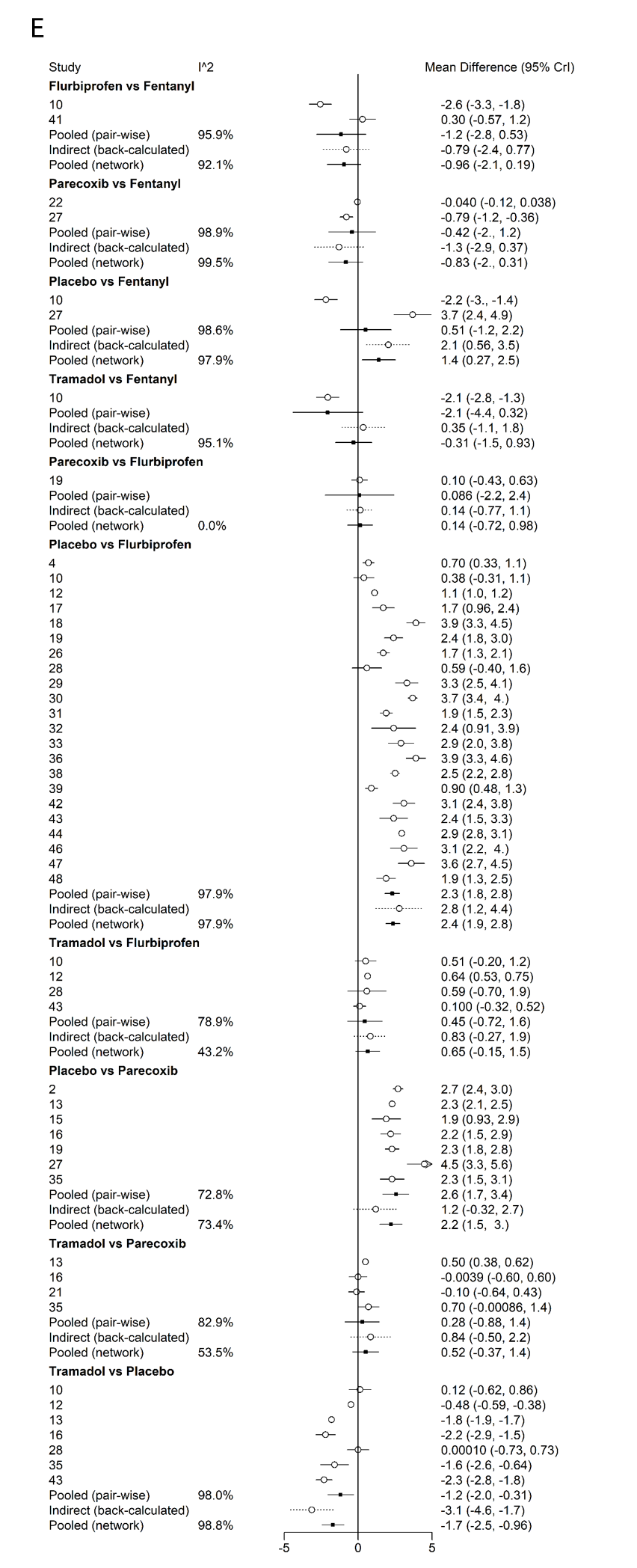

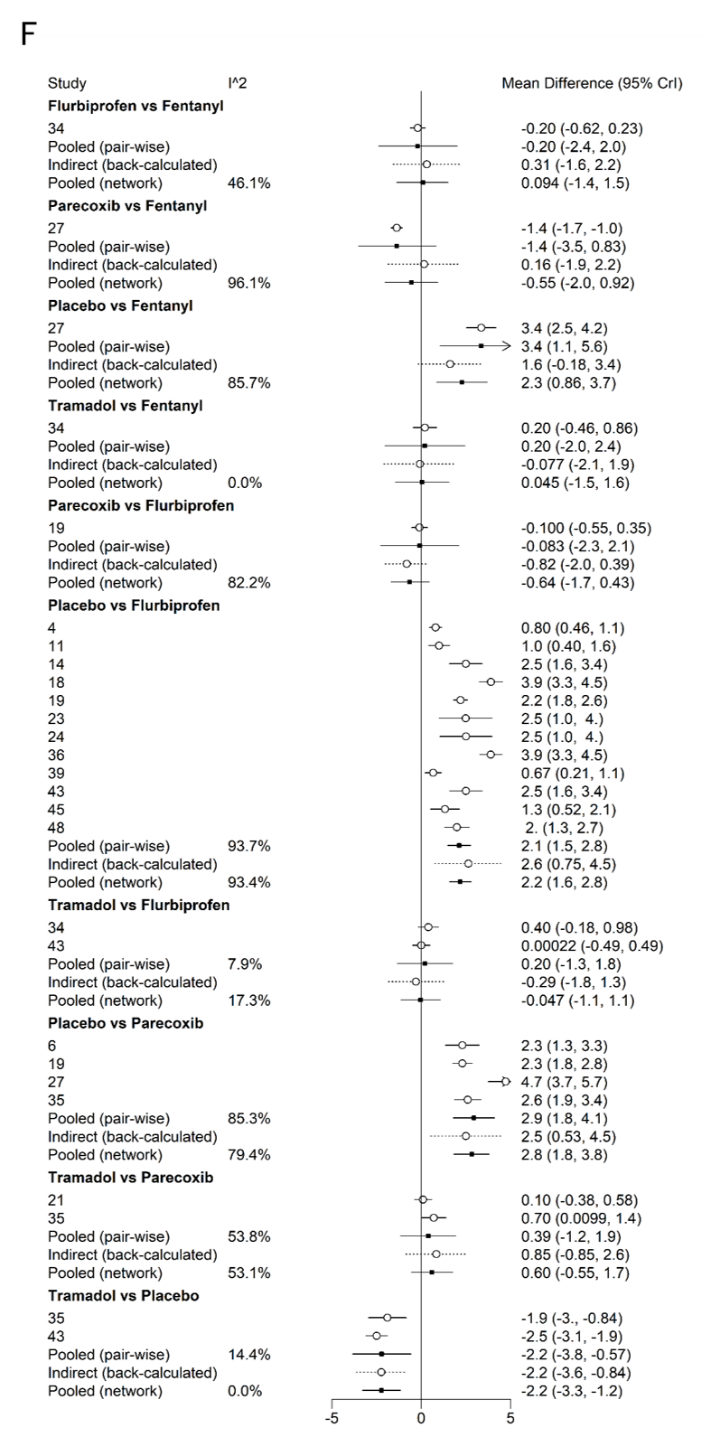


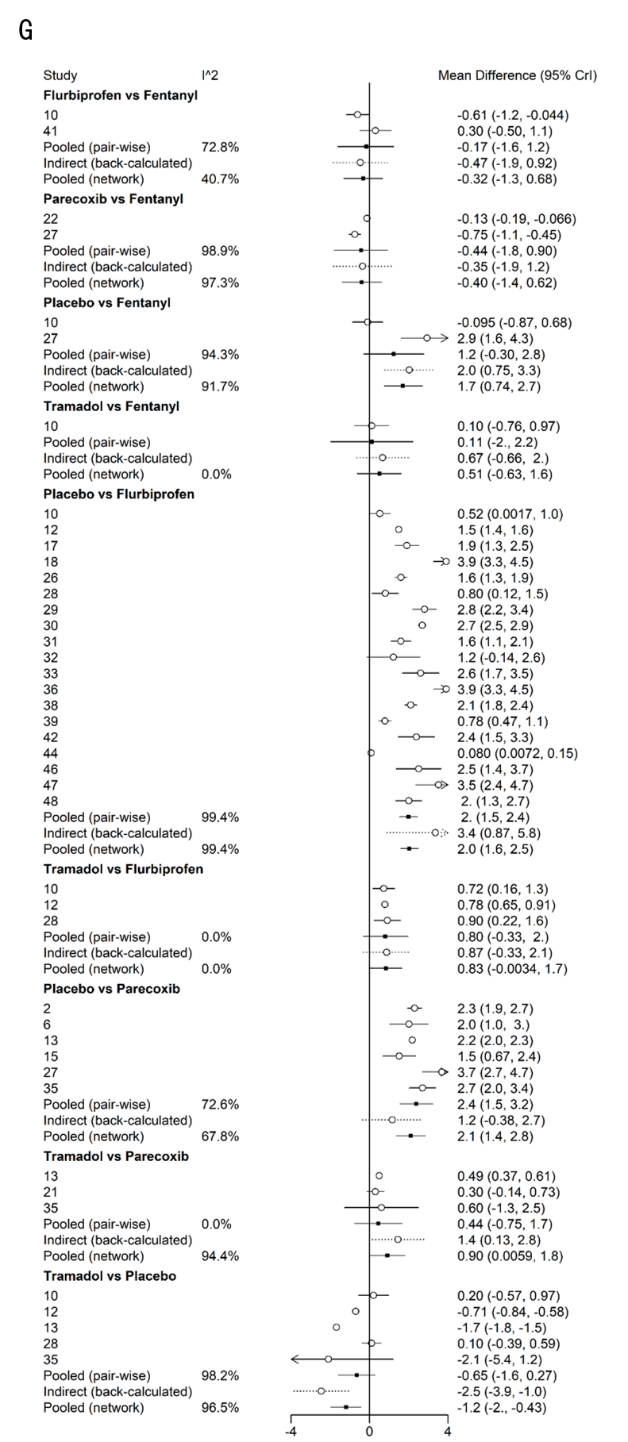

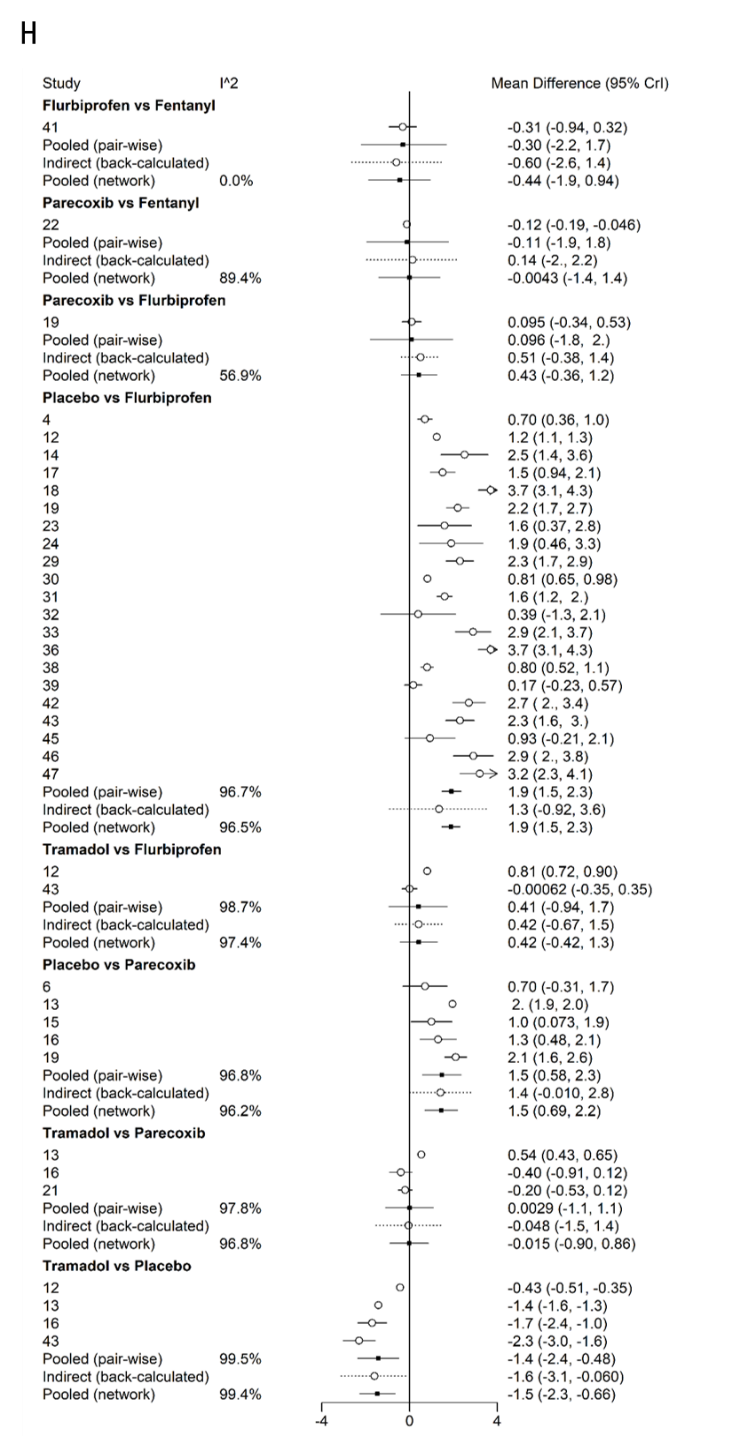


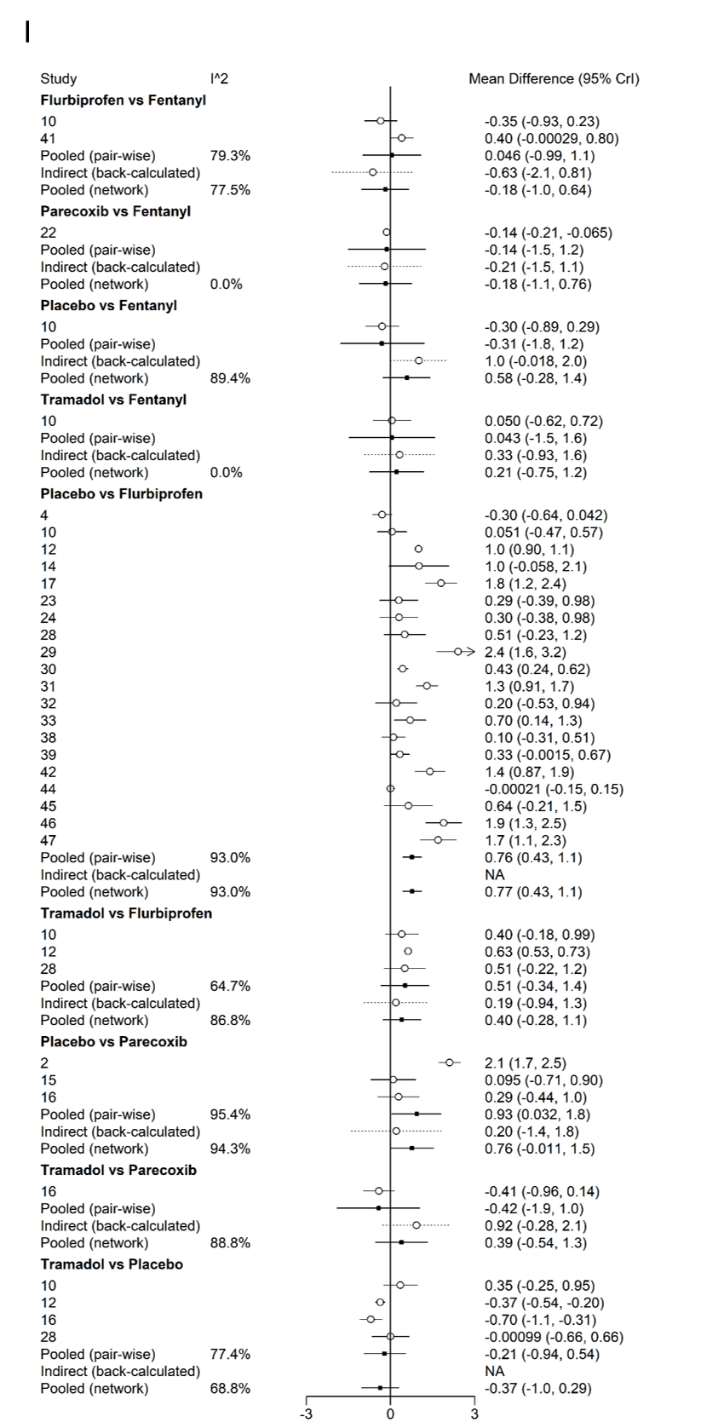

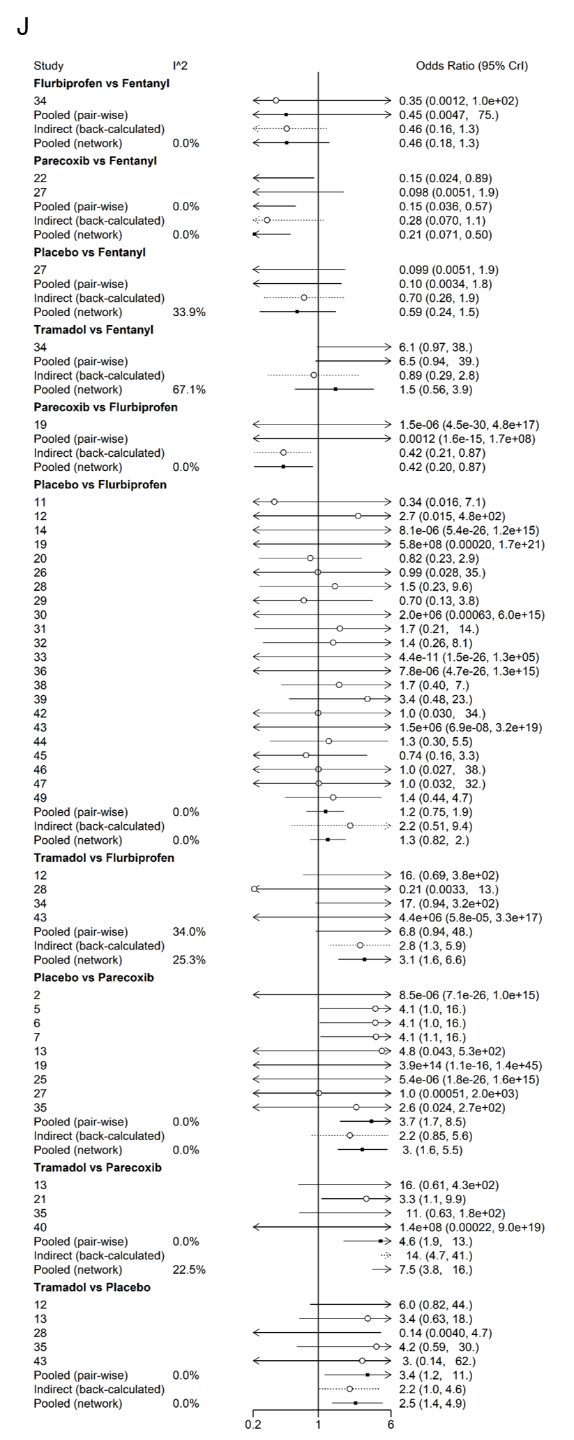


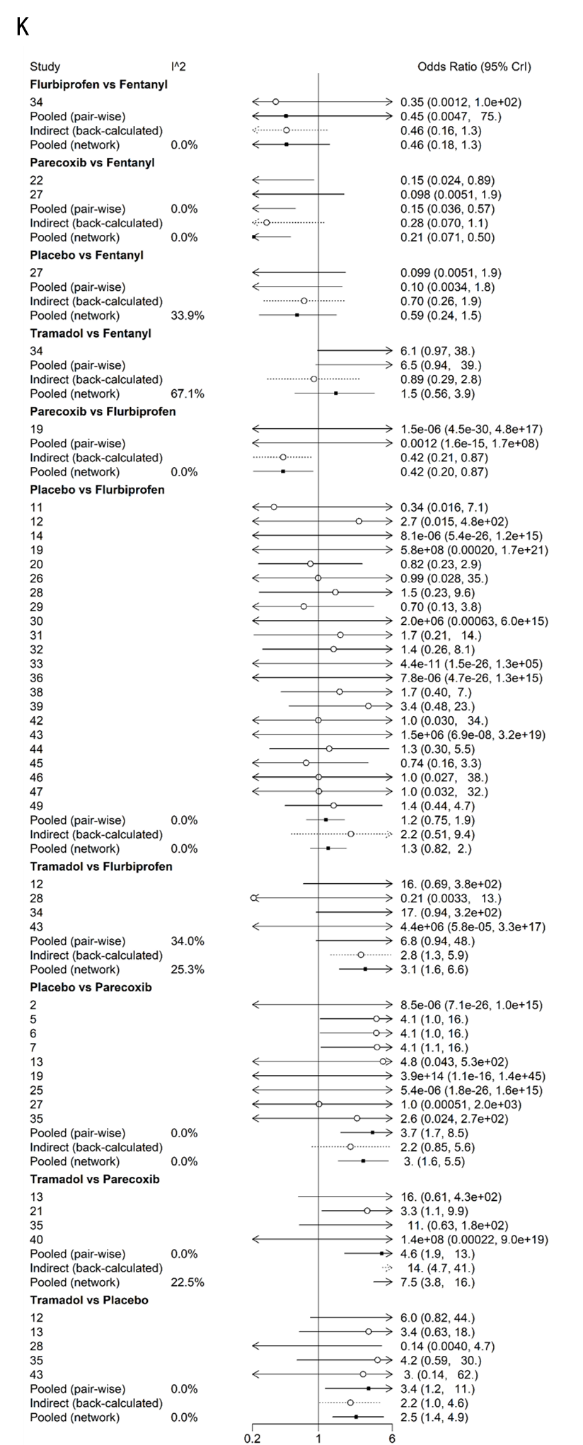

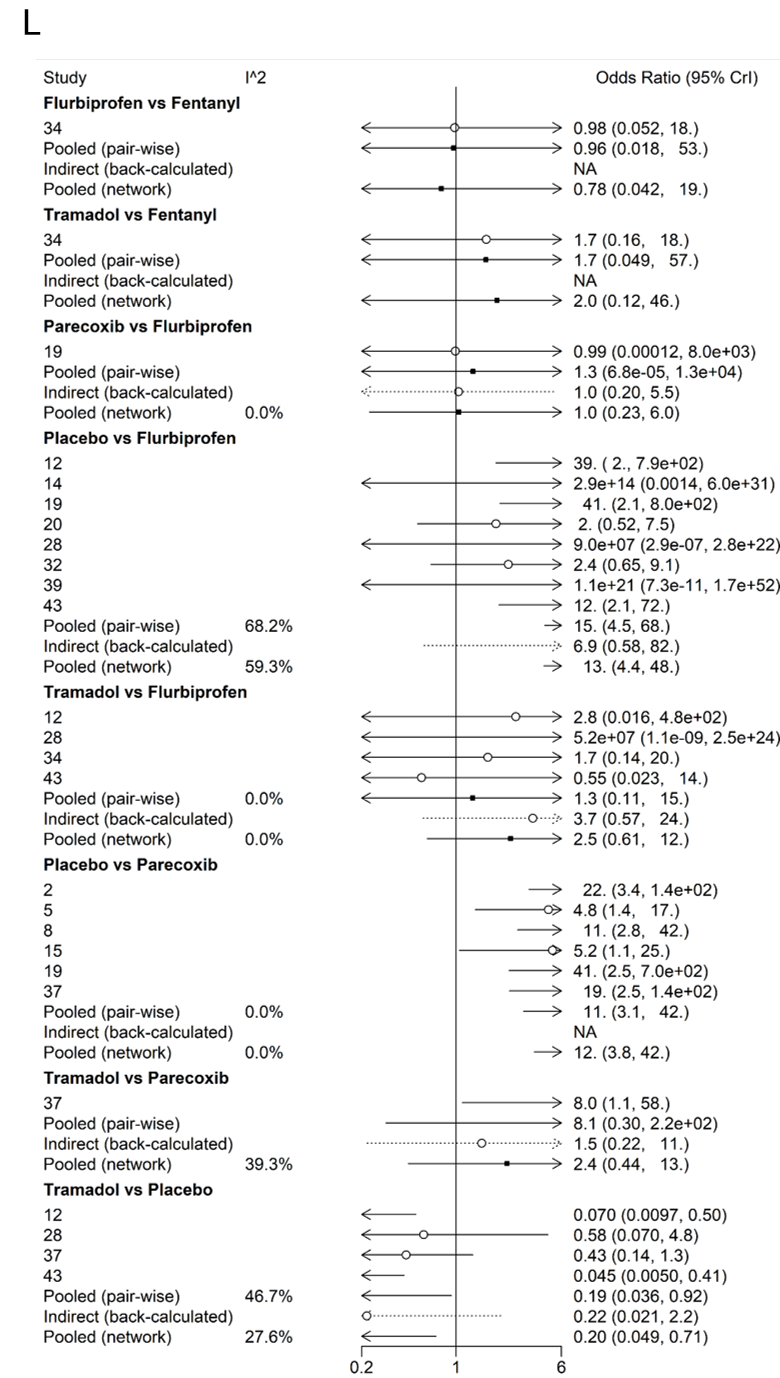


Supplemental Figure 4: The funnel plot of enrolled trials.

A, pain scores at postoperative 0 h. B, pain scores at postoperative 0.5 h. C, pain scores at postoperative 1 h. D, pain scores at postoperative 2 h. E, pain scores at postoperative 4 h. F, pain scores at postoperative 6 h. G, pain scores at postoperative 8 h. H, pain scores at postoperative 12 h. I, pain scores at postoperative 24 h. J, incidence of total adverse events. K, incidence of postoperative nausea and vomiting. L, incidence of agitaion after surgery.


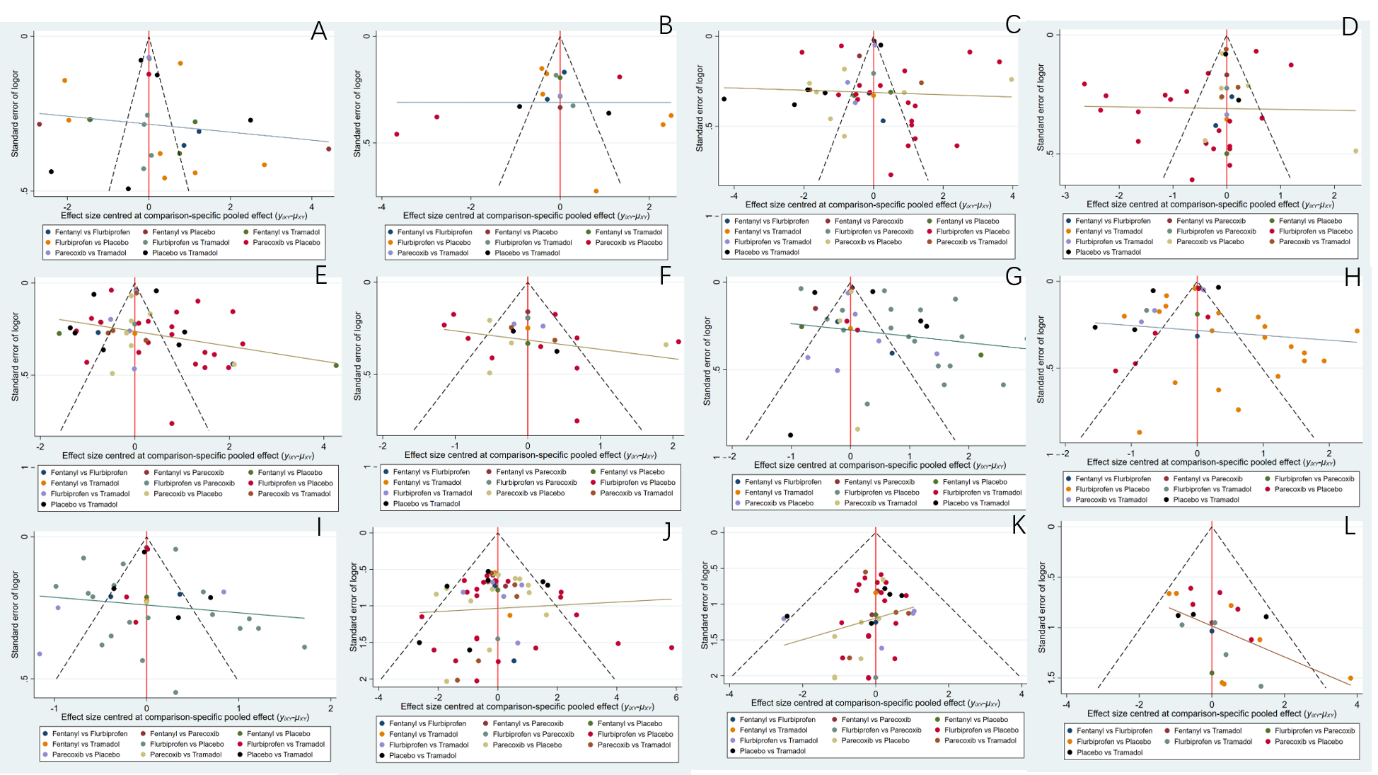

Supplement: Supplementary file 1 [file Data_Sheet_1.docx]
